# The proteomic response of the reef coral *Pocillopora acuta* to experimentally elevated temperatures

**DOI:** 10.1371/journal.pone.0192001

**Published:** 2018-01-31

**Authors:** Anderson B. Mayfield, Yi-Jyun Chen, Chi-Yu Lu, Chii-Shiarng Chen

**Affiliations:** 1 Khaled bin Sultan Living Oceans Foundation, Annapolis, MD, United States of America; 2 Taiwan Coral Research Center, Checheng, Pingtung, Taiwan; 3 Department of Biochemistry, College of Medicine, Kaohsiung Medical University, Kaohsiung, Taiwan; 4 Center for Research Resources and Development, Kaohsiung Medical University, Kaohsiung, Taiwan; 5 Department of Marine Biotechnology and Resources, National Sun Yat-Sen University, Kaohsiung, Taiwan; 6 Graduate Institute of Marine Biotechnology, National Dong-Hwa University, Checheng, Pingtung, Taiwan; Instituto Nacional de Salud Pública, MEXICO

## Abstract

Although most reef-building corals live near the upper threshold of their thermotolerance, some scleractinians are resilient to temperature increases. For instance, *Pocillopora acuta* specimens from an upwelling habitat in Southern Taiwan survived a nine-month experimental exposure to 30°C, a temperature hypothesized to induce stress. To gain a greater understanding of the molecular pathways underlying such high-temperature acclimation, the protein profiles of experimental controls incubated at 27°C were compared to those of conspecific *P*. *acuta* specimens exposed to 30°C for two, four, or eight weeks, and differentially concentrated proteins (DCPs) were removed from the gels and sequenced with mass spectrometry. Sixty unique DCPs were uncovered across both eukaryotic compartments of the *P*. *acuta-*dinoflagellate (genus *Symbiodinium*) mutualism, and *Symbiodinium* were more responsive to high temperature at the protein-level than the coral hosts in which they resided at the two-week sampling time. Furthermore, proteins involved in the stress response were more likely to be documented at different cellular concentrations across temperature treatments in *Symbiodinium*, whereas the temperature-sensitive host coral proteome featured numerous proteins involved in cytoskeletal structure, immunity, and metabolism. These proteome-scale data suggest that the coral host and its intracellular dinoflagellates have differing strategies for acclimating to elevated temperatures.

## Introduction

Given the significant anthropogenic pressures currently placed upon coral reef ecosystems [1], there is an urgent need to understand how corals will respond to the elevated temperatures that will characterize their habitats in the coming years [2–3]. Although corals tend to exist at the upper threshold of their thermotolerance [4], some can tolerate dramatic fluctuations [5–6] and increases [7] in temperature. Oliver and Palumbi [8] hypothesized that corals living in thermodynamically extreme environments may be more adept at surviving future increases in temperature, and such was confirmed in Taiwan [9]; *Pocillopora acuta* (misidentified as *P*. *damicornis* in the cited reference) specimens from a reef (“Houbihu”) characterized by highly variable temperatures due to episodic upwelling in Taiwan’s southernmost embayment, Nanwan, survived a nine-month exposure to 30°C, a temperature they experience for only several hours in a typical year and therefore one hypothesized to induce stress.

Since molecular biology-driven approaches have revolutionized our understanding of the fundamental biology of anthozoan-dinoflagellate endosymbioses (e.g., [10–18]), as well as how these environmentally sensitive mutualisms respond to environmental change (e.g., [19–28]), a next generation sequencing-based transcriptome profiling approach (RNA-Seq) was previously taken to attempt to uncover how the aforementioned pocilloporid corals from upwelling reefs of Southern Taiwan acclimated to such dramatic and prolonged increases in temperature [29]. The expression levels of numerous mRNAs in both compartments (coral host and dinoflagellate endosymbionts of the genus *Symbiodinium*) were affected by both short- (2-week) and long-term (36-week) elevated temperature exposure. From this transcriptome-scale dataset, the authors surmised that effective modulation of protein turnover might have enabled such high-temperature acclimation; indeed, protein turnover had already been hypothesized by other coral reef researchers to be a key cellular process underlying the capacity for certain coral species to acclimate to high temperatures [30–31].

Although protein homeostasis/turnover may ultimately be implicated in coral acclimation to high temperatures, it is possible that the concentrations of the proteins encoded by the differentially expressed genes (DEGs) uncovered in the aforementioned transcriptomic analysis did not actually differ between corals incubated at the control and high-temperature treatments. In fact, the degree of congruency between mRNA expression and protein concentration was markedly low in another pocilloporid, *Seriatopora hystrix*, exposed to either stable (26°C) or variable temperatures (23–29°C over a 6-hr period) for seven days [32]; only 2 of the 167 differentially concentrated proteins (DCPs) were associated with an mRNA that was also differentially expressed between treatments. This result highlights the role of post-transcriptional processes in *S*. *hystrix*, and alternative splicing, in fact, was found to vary dramatically across temperature treatments based on proteome profiling data from those same *S*. *hystrix* samples [32].

Given the need to better understand the molecular mechanisms underlying the ability of certain corals to acclimate to elevated temperatures, as well as the previously documented lack of congruency between mRNA expression and protein abundance in reef corals and their in *hospite Symbiodinium* populations, proteins co-extracted from the same *P*. *acuta* biopsies from which transcriptomes were previously sequenced [29] were electrophoresed across two dimensions (2D) herein. Proteins that were uniquely translated by samples of one treatment (i.e., “uniquely synthesized proteins” [USPs]), or, alternatively, documented at different concentrations between treatments (i.e., DCPs), were isolated and sequenced with mass spectrometry (MS). MS-based approaches have proven useful in identifying proteins responsive to extreme high temperature events over short-term time periods (1–2 days) in reef corals [33], and it was hypothesized herein that 1) a low degree of congruency would be documented between mRNA expression and protein concentration in samples of the two-week sampling time (the transcriptomes of the four-and eight-week samples were not sequenced.), and 2) a molecular mechanism underlying high-temperature acclimation could be uncovered for this widely distributed, model reef-builder using the proteome-scale data generated. Since the *Symbiodinium* compartment showed a more pronounced mRNA-level response upon incubation at elevated temperatures than the coral hosts in which they resided [29], it was also hypothesized that high temperature exposure would more dramatically affect their proteomes than those of their anthozoan hosts.

## Materials and methods

### The experiment

The details of this long-term temperature experiment can be found in another work [9]. Briefly, *P*. *acuta* nubbins created from colonies originally collected from Houbihu, Taiwan (21°56’18.01”N, 120°44’45.54”E) under Kenting National Park permit 0992900842 (to Dr. Tung-Yung Fan) were exposed to either a control temperature of 27°C or an elevated one of 30°C (n = 3 replicate coral reef mesocosms for each of the two treatments). Despite the fact that upwelling events routinely occur in the boreal summer at Houbihu, corals of Houbihu experience only ephemeral periods of 30°C *in situ* [34]; this temperature was therefore hypothesized to induce stress. Nubbins (1–2 per mesocosm x 3 mesocosms/treatment x 2 treatments) were collected after 0, 2, 4, 8, 24, or 36 weeks of treatment exposure, and the physiological [9] and mRNA expression (i.e., RNA-Seq) data [29] were described previously. Corals acclimated to the elevated temperature regime; however, the gastrodermal tissue layer became thicker in response to a long-term (36-week) exposure to 30°C [9], and *Symbiodinium* ubiquitin ligase mRNA expression increased dramatically upon a 2-week exposure to this temperature [29].

### Protein extraction, 2D gel electrophoresis, and MS

Proteins were extracted with TRIzol® (Life Technologies) as described previously [9] from one randomly selected nubbin from each of three replicate tanks at each of the two temperatures at the two-, four-, and eight-week sampling times (n = 18 protein extractions). Additional details of the protein extraction protocol can be found in the S1 File.

Proteins (~150 μg) were electrophoresed across 2D as described previously [35]. For each sampling time, a control-temperature protein sample was electrophoresed at the same time as a high-temperature sample, and three replicate control vs. high-temperature pairs were analyzed for each treatment and sampling time (18 gels in total were run.). Each of the triplicate gels for each treatment x time group featured a protein extracted from a nubbin generated from a different source coral colony (n = 3 biological replicates per sampling time). As the aforementioned book chapter [35] is not freely available except on ABM’s personal website (coralreefdiagnostics.com), the details have been reiterated in the S1 File. Briefly, isoelectric point (pI) and molecular weight (kDa) were the first and second dimensions, respectively.

**Fig 1:**
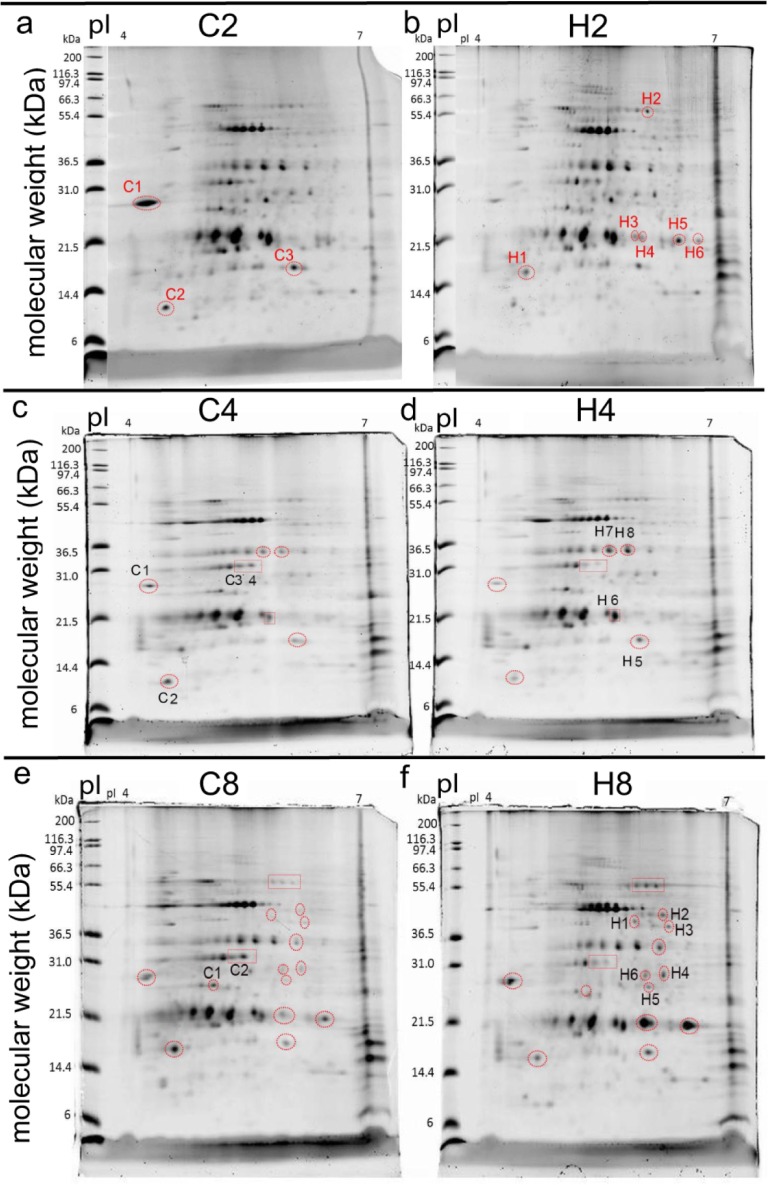
Two-dimensional gel electrophoresis of proteins from representative control and high- temperature coral samples after two, four, and eight weeks of treatment exposure. Encircled and labeled protein spots were 1) determined to be differentially concentrated between temperature treatments within a sampling time with image analysis software, 2) removed from the gels, 3) purified, 4) digested with trypsin, and 5) submitted for sequencing by nano-liquid chromatography+mass spectrometry as described in the main text and the S1 File. The isoelectric point (pI) and molecular weight (in kilodaltons [kDa]) were the 1^st^ and 2^nd^ dimensions, respectively. Only labeled spots were removed; in certain cases at the four- and eight-week sampling times, matched (i.e., same pI and molecular weight) spots have been encircled in gels of both treatments to emphasize their differential concentrations, yet they were not sequenced for reasons discussed in the main text. “C2” and "H2" = control and high-temperature samples at the two-week sampling time, respectively. “C4” and "H4" = control and high-temperature samples at the four-week sampling time, respectively. “C8” and "H8" = control and high-temperature samples at the eight-week sampling time, respectively.

Unique and differentially concentrated protein spots were identified with ImageQuant™ TL software provided with the Typhoon Trio™ scanner (GE Healthcare) used to image the gels (described in detail in the S1 File), and only protein spots that were found by ImageQuant TL to differ in concentration in all three pairs of control vs. high temperature gels for each sampling time were targeted for removal from the representative gel. Proteins that were unique to the proteomes of samples of the control and high-temperature treatments at a particular sampling time were removed from a representative control and high temperature gel, respectively. When no USPs were uncovered, DCPs were instead targeted; in these cases, the protein spot was always removed from the gel in which its concentration was higher. Nine, eight, and eight spots were removed from each representative pair of gels at the two-, four-, and eight-week sampling times, respectively. In certain cases, not every DCP spot was removed from the representative gel due to certain spots being over-saturated; these were likely comprised of a complex mix of a plethora of proteins, and the spot intensity could consequently not be traced to the concentration of a unique protein. Some such protein spots have been encircled in Fig 1 to highlight their differential abundance, though only those given identification codes were removed from the gels.

USP (n = 8) and DCP (n = 17) spots (Table 1) were removed from the representative gels, digested with trypsin, and prepared for sequencing with MS as described previously [35]. Given the potential inaccessibility of this book chapter, the details are presented again in the S1 File. Digested peptides (2 μl) were sequenced on a nano-liquid chromatography system and detected by an LTQ Orbitrap Discovery Hybrid Fourier Transform Mass Spectrometer (Thermo-Fisher Scientific) at a resolution of 30,000 coupled with a nanospray source that was executed in positive ion mode. The nano-UPLC system (“nanoACQUITY”) was purchased from Waters, as were the desalting (Symmetry C18, 5 μm x 180 μm x 20 mm) and analytical (BEH C18, 1.7 μm x 75 μm x 150 mm) columns. The peptide eluate from the column was directed to the nanospray source, and the MS was operated in data-dependent mode.

**Table 1:** Summary of two-dimensional gel electrophoresis results.

Comparison (Fig)	#C>H spots removed	#C>H USP spots	#C>H DCP spots	#C>H DCPs identified	# C>H host DCPs	# C>H Sym DCPs	#H>C spots re-moved	#H>C USP spots	#H>C DCP spots	#H>C DCPs identi-fied	#H>C host DCPs	#H>C Sym DCPs	Total host/ Sym DCPs
C2 vs. H2 (Fig 2)	3	0	3	13	7	5	6	4	2	25	8	10	15/15^a^
C4 vs. H4 (Fig 3)	4	0	4	7	6	1	4	0	4	3	2	1	8/2^b^^,^^c^
C8 vs. H8 (Fig 4)	2	1	1	6	2	4	6	3	3	16	12	2	14/6^c^
Total (Fig 5)	9	1	8	26	15	10	16	7	9	44	22	13	37/23

In certain cases, the total number of differentially concentrated proteins (DCPs) does not equal the sum of the host coral and *Symbiodinium* (Sym) DCPs; this is due to either 1) certain proteins being of bacterial origin or 2) the inability to assign a compartment of protein origin in some cases. The five proteins that were documented at different concentrations between temperature treatments at multiple sampling times were counted only at the earlier of the two times at which they were sequenced, leading to a total number of unique host+Sym DCPs of 60 (Fig 5D). For a graphical representation of a portion of the data in this table, please see Fig 6A. C = control treatment. H = high temperature treatment. The numbers after these treatment abbreviations in the “Comparison” column reflect the sampling times (e.g., C2 = control samples of the two-week sampling time). USP = uniquely synthesized protein.

^a^host/Sym DCP ratio significantly lower (*z*-test, *p*<0.05) than the host/Sym mRNA transcript ratio (1.9±0.4 [std. dev.]).

^b^host/Sym DCP ratio significantly higher (*z*-test, *p*<0.05) than the host/Sym mRNA transcript ratio.

^c^Host/Sym DCP ratio differs slightly from that of the respective figure since only unique DCPs were considered in this table; in contrast; all DCPs have been depicted in the compartmental breakdown pie graphs of Fig 4 (10/2 ratio) and 5 (15/7 ratio).

### Data analysis

Data files from the spectrometer (.MGF) were used directly as queries of the *P*. *acuta-Symbiodinium* transcriptome with “MS-SCAN.” This feature of the transcriptome server was described previously [29], and the program can be accessed on the following website: http://symbiont.iis.sinica.edu.tw/coral_pdltte/static/html/index.html#mscan. Details of the MS-SCAN program can be found in the S1 File, as well as in Kim and Pevzner [36]. The.MGF files produced herein (n = 25) can be downloaded from the following page: http://symbiont.iis.sinica.edu.tw/coral_pdltte/static/html/index.html#stat.MGF files were directly uploaded onto the MS-SCAN program hosted on the transcriptome server, and the digestion enzyme was set to trypsin. In addition to the default parameters for protein identification described in the S1 File and in Kim and Pevzner [36], only those proteins that featured either 1) 15 or more amino acids (AA) sequenced or 2) two peptides mapping to the same reference protein (whose collective length summed to 15 or more AA) were considered. When a protein was documented at relatively higher concentrations in both control and high-temperature gels at the same sampling time, it was discarded from the analysis. These peptide length/number criteria are more stringent than what are commonly employed in the vertebrate (e.g., [37]) and reef coral research ([33,38]) fields, where, furthermore, only one biological replicate is typically used per treatment (but see Weston et al. [39]); however, for those interested in using less stringent peptide inclusion criteria, both peptide length (default = 15 AA) and count (default = 2 peptides mapping to the same reference protein) thresholds can be reduced on the MS-SCAN program implemented on our interactive transcriptome website.

For all peptides fulfilling our criteria, the respective mRNA sequences from the top hit contigs derived from the MS-SCAN analysis of the *P*. *acuta-Symbiodinium* transcriptome were BLASTed against 1) the *Stylophora pistillata* genome and 2) the *Symbiodinium* (clade B1) genome (both housed on the NCBI database) to verify protein identity, and proteins were assigned a compartment of origin: unknown, bacteria, host coral, or *Symbiodinium*. When a contig aligned significantly (*e*<10^−5^) to a functionally characterized protein, the respective cellular process (e.g., metabolism) was recorded (if known). The top hit contig sequence, rather than the peptide sequence itself, was used as the BLAST query due to the short nature of the latter (mean length = 20±10 [std. dev. for this and all error terms henceforth] AA) and the consequent inability to generate significant alignments in many cases; that being said, for those peptides >30 AA, the MS-SCAN-derived peptide sequence itself was additionally BLASTed against both the *Symbiodinium* and *S*. *pistillata* genomes to corroborate findings from 1) MS-SCAN and 2) the mRNA-BLAST, and, in the cases when the peptide itself aligned significantly to a presumed homolog in the NCBI database (*e*<10^−5^), the first and second NCBI accession numbers in the S1–S3 Tables correspond to the top mRNA-BLAST hit and the top peptide-BLAST hit, respectively.

Expression data (fragments per kilobases mapped) were obtained from the transcriptome server (http://symbiont.iis.sinica.edu.tw/coral_pdltte/static/html/index.html#stat) for each of the mRNAs hypothetically encoding the DCPs uncovered to understand the degree of congruency between mRNA expression and protein concentration for samples of the two-week sampling time only. Details of this analysis can be found in the S1 File. *z*-tests were used to determine whether the compartmental breakdown of the DCPs deviated significantly from the host/*Symbiodinium* mRNA ratio of 1.9±0.4 determined previously [29]. Two-sample proportion tests were used to determine whether certain cellular processes (e.g., stress response) in the differentially concentrated proteomes were over-represented relative to their proportional abundance in the transcriptome [29]; this and all other proportion-based analyses were only performed for those cellular processes in which ≥2 DCPs were identified. *X*^*2*^ tests were used to ascertain whether cellular processes were equally represented across sampling times, as well as whether the host/*Symbiodinium* DCP ratio differed significantly over time. All statistical analyses were performed with JMP® (ver. 12.0.1), and an alpha level of 0.05 was set for all aforementioned tests.

## Results

### Overview of data

Summaries of the 2D+MS analysis and sequenced peptides for all three sampling times can be found in Tables 1 and 2, respectively, and detailed information on the DCPs uncovered at the two-, four-, and eight-week sampling times (e.g., top hit contigs, accession numbers, peptide lengths, and protein coverage [%]) can be found in the S1 Table, S2 Table and S3 Table, respectively; the respective peptide sequences and associated cellular pathways are located in the S4 Table, S5 Table and S6 Table, respectively.

**Table 2:** Summary of all unique host coral (*Pocillopora acuta*) and *Symbiodinium* differentially concentrated proteins (DCPs) uncovered across temperature treatments.

time spot	protein	compartment	cellular process	concentration
**TWO WEEKS (n = 38)**				
C1-2	pentraxin^a^	host	immunity	control>high-temp.
C1	transcription factor death-induced obliterator-1	host	transcription factor	control>high-temp.
C1	protein kinase UbiB	*Symbiodinium*	protein kinase	control>high-temp.
C1	STI1-like protein	*Symbiodinium*	stress response	control>high-temp.
C1	myosin-6^b^	*Symbiodinium*	actin-based motility	control>high-temp.
C2	WSC domain	host	unknown	control>high-temp.
C2	cadherin EGF LAG seven-pass G-type receptor 2^a^	host	receptor/signaling	control>high-temp.
C2	pre-mRNA splicing factor SLU7-A^b^	*Symbiodinium*	gene expression/splicing	control>high-temp.
C3	hypothetical protein	host	unknown	control>high-temp.
C3	Pao retrotransposon peptidase	host	unknown/various	control>high-temp.
C3	spectrin alpha chain^a^	host	cytoskeleton	control>high-temp.
C3	adenylate kinase	*Symbiodinium*	protein kinase	control>high-temp.
C3	hypothetical protein	unknown	unknown	control>high-temp.
H1	hypothetical protein	host	unknown	high>control temp.
H1, H3	low-density lipoprotein receptor-related protein 4^a^	host	receptor/transport	high>control temp.
H1	serine/arginine repetitive matrix protein 1^a^	host	gene expression/splicing	high>control temp.
H1	Rec10 / Red1	*Symbiodinium*	meiosis	high>control temp.
H1	VWA domain-containing protein	bacteria	unknown	high>control temp.
H2	hypothetical protein	host	unknown	high>control temp.
H2	histone-lysine N-methyltransferase SETD1B-like^a^	host	gene expression/splicing	high>control temp.
H2	ribulose-1,5-bisphosphate carboxylase/oxygenase^b^	*Symbiodinium*	photosynthesis	high>control temp.
H2-3	protein with DNAJ and WW domains*	*Symbiodinium*	stress response	high>control temp.
H2	hypothetical protein	unknown	unknown	high>control temp.
H3	golgin subfamily B member 1^a^	host	unknown	high temp. only
H3	peptidylprolyl isomerase D*	*Symbiodinium*	stress response, various	high temp. only
H3	hypothetical protein	unknown	unknown	high temp. only
H3	hypothetical protein	unknown	unknown	high temp. only
H4	E3 ubiquitin protein ligase	*Symbiodinium*	stress response	high temp. only
H4	hypothetical protein	unknown	unknown	high temp. only
H4	hypothetical protein	unknown	unknown	high temp. only
H5-6	beta-gamma crystallin	host	stress response	high temp. only
H5	sacsin^a^	host	stress response	high temp. only
H5	hypothetical protein	unknown	unknown	high temp. only
H6	ribosome biogenesis protein NSA2-like^b^	*Symbiodinium*	ribosome	high temp. only
H6	pentatricopeptide repeat-containing protein*	*Symbiodinium*	unknown	high temp. only
H6	voltage-dependent T-type calcium channel subunit α-1H	*Symbiodinium*	transport	high temp. only
H6	nucleolar protein of 40 kDa	*Symbiodinium*	unknown	high temp. only
H6	peptidylprolyl isomerase D* (differs from paralog in spot H3)	*Symbiodinium*	stress response, various	high temp. only
**FOUR WEEKS (n = 12)**				
C1	pentraxin^a^^,^^c^	host	immunity	control>high-temp.
C1	trichohyalin^d^	host	structural	control>high-temp.
C1	plexin^a^	host	signal transduction/reception	control>high-temp.
C2	avidin	host	reproduction	control>high-temp.
C2	hypothetical protein	host	unknown	control>high-temp.
C3	chloroplast oxygen-evolving enhancer^b^	*Symbiodinium*	photosynthesis	control>high-temp.
C4	actin	host	cytoskeleton	control>high-temp.
C4	actin (different from previous paralog)	host	cytoskeleton	control>high-temp.
H7	RNA-directed DNA polymerase from mobile element jockey-like^a^	host	gene expression/splicing	high>control temp.
H7-8	peridinin-chlorophyll A binding protein^b^	*Symbiodinium*	photosynthesis	high>control temp.
H8	NF-kappa-B inhibitor-like protein 1 isoform X2^a^	host	immunity	high>control temp.
H8	hypothetical protein^c^	host	unknown	high>control temp.
**EIGHT WEEKS (n = 25)**				
C1	rho GDP-dissociation inhibitor 1-like^a^	host	protein homeostasis	control temp. only
C1	hypothetical protein	*Symbiodinium*	unknown	control temp. only
C1	fucoxanthin-chlorophyll a-c binding protein F^b^	*Symbiodinium*	photosynthesis	control temp. only
C1	hypothetical protein^b^	*Symbiodinium*	unknown	control temp. only
C1	metal transporter Nramp3	*Symbiodinium*	transport	control temp. only
C2	centromere-associated protein E-like isoform X1	host	cell cycle	control>high temp.
H1	trichohyalin^e^	host	structural	high temp. only
H1	hypothetical protein	*Symbiodinium*	unknown	high temp. only
H1	hypothetical protein	unknown	unknown	high temp. only
H1	hypothetical bacterial protein^c^	bacteria	unknown	high temp. only
H2	abhydrolase^a^	host	metabolism	high temp. only
H2	chromodomain-helicase-DNA-binding protein 1-like^a^^,^^f^	host	DNA repair/transcription	high temp. only
H2	hypothetical protein^b^	*Symbiodinium*	unknown	high temp. only
H3	retrovirus-related Pol polyprotein from transposon 17.6^a^	host	unknown	high temp. only
H3	guanine nucleotide-binding protein G(I)/G(S)/G(T) subunit beta-1^a^	host	signaling	high temp. only
H3	stabilizer of axonemal microtubules 2-like^a^	host	cytoskeleton	high temp. only
H3	glycerol-3-phosphate dehydrogenase^a^	host	metabolism	high temp. only
H3	concanavalin A-like lectin/glucanase superfamily	host	cell membrane binding	high temp. only
H4	glycosyltransferase-like domain-containing protein 1	host	metabolism	high>control temp.
H4	trichohyalin-like	host	cell structure	high>control temp.
H4	protein w/ DNAJ and WW domains^c^	*Symbiodinium*	stress response	high>control temp.
H4	hypothetical protein	unknown	unknown	high>control temp.
H5	peroxiredoxin-6-like	host	stress response	high>control temp.
H5	hypothetical protein	host	unknown	high>control temp.
H6	endonuclease	host	DNA repair	high>control temp.

For additional details of the two- (n = 38), four- (n = 12), and eight-week (n = 25) DCPs, please see the S1 Table, S2 Table and S3 Table, respectively. For the respective amino acid sequences, please see the S4 Table, S5 Table and S6 Table, respectively.

*congruency between mRNA expression and protein concentration. temp. = temperature.

^a^Protein identity corroborated by directly BLASTing peptide sequence against the *Stylophora pistillata* genome.

^b^Protein identity corroborated by directly BLASTing peptide sequence against the *Symbiodinium* (clade B1) genome.

^c^Also identified at the two-week sampling time.

^d^Also identified at the eight-week sampling time.

^e^Also identified at the four-week sampling time.

^f^Protein concentration affected by temperature treatment in a study undertaken with the con-familial coral *Seriatopora hystrix* [32].

### Two-week sampling time

**Fig 2:**
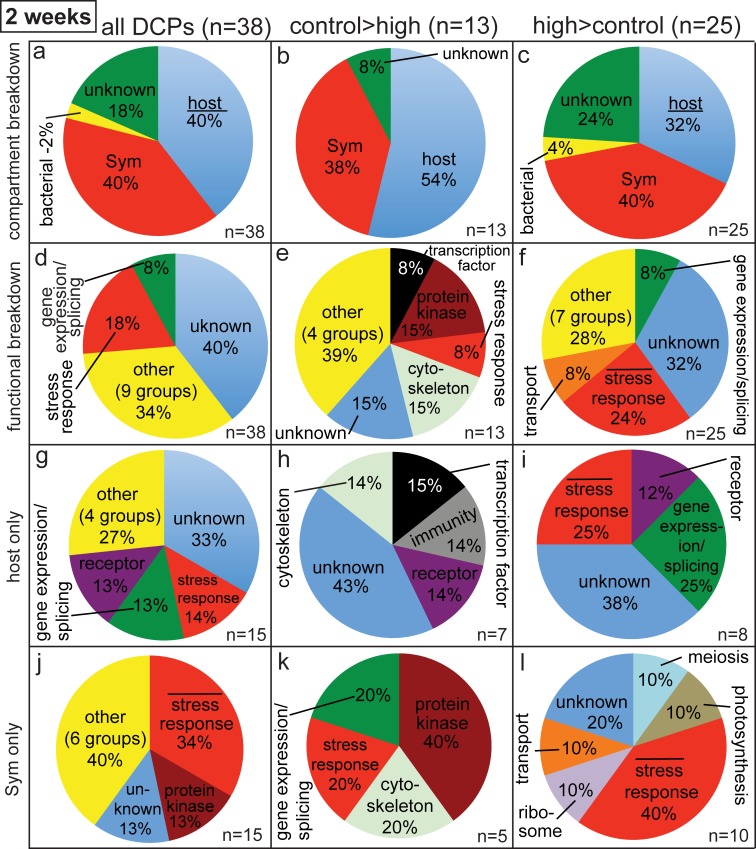
Pie graphs showing breakdown of differentially concentrated proteins (DCPs) between control (C2) and high (H2) temperature samples of the two-week sampling time. Three and six protein spots were found to be more highly concentrated in the C2 (i.e., C>H) and H2 (H>C) proteomes, respectively, and 13 C>H and 25 H>C DCPs were identified from these nine spots. When the ratio of host/*Symbiodinium* (Sym) DCPs in panels a and c was significantly lower (*z*-test, *p*<0.05) than the *Pocillopora acuta*/Sym mRNA ratio of 1.9 (i.e., a 65/35% host coral/Sym ratio), the word “host” has been underlined. When the cellular process “stress response” was over-represented in the proteomes of the host coral (g-i) and Sym (j-l) relative to the host coral (6% of the *P*. *acuta* host transcriptome) and Sym (4% of the Sym transcriptome) transcriptomes [29], respectively (2-sample proportion test, *p*<0.05), a bar has been inserted over the term “stress response”.

Upon electrophoresing proteins across 2D, there were four protein spots consistently present only in the high-temperature gels of the two-week sampling time (Fig 1B). All four were removed from the representative gel and sequenced, as were three and two additional protein spots documented at higher concentrations in the C2 (Fig 1A) and H2 (Fig 1B) gels, respectively (n = 9 sequenced spots). Upon querying the *P*. *acuta-Symbiodinium* transcriptome with MS-SCAN, 38 unique proteins were sequenced across these nine spots, 13 and 25 of which were at higher concentrations in the control and high-temperature gels, respectively (Table 2, S1 Table and S4 Table). Eight of these DCPs (Fig 2A) were either of bacterial origin or could not be assigned a compartment of origin or function; these proteins are not discussed further herein. There were equal numbers of coral and *Symbiodinium* DCPs (n = 15); given that the host comprises ~60–65% of the biomass of *P*. *acuta* [29], this represents a statistically significant over-representation of *Symbiodinium* proteins (host/*Symbiodinium* protein ratio of 1 < host/*Symbiodinium* mRNA ratio of 1.9±0.4; *z*-test, *p*<0.05). A relative over-representation of *Symbiodinium* DCPs was also documented for the proteins whose concentrations were higher in high-temperature samples (Fig 2C; *z*-test, *p*<0.05) but not for those more highly concentrated by the control samples (Fig 2B; *z*-test, *p*>0.05).

In terms of the functional breakdown of the two-week sampling time DCPs (Fig 2D–2L), the two most represented categories were the stress response and gene expression/splicing; 18% of all two-week DCPs (n = 7/38) were involved in the former process (Fig 2D). Although “stress response” was not over-represented relative to its proportional abundance in the composite *P*. *acuta-Symbiodinium* transcriptome (5%) for proteins more highly concentrated by the control coral samples (8%; Fig 2E), it was for those proteins more highly concentrated by the high- temperature samples (Fig 2F; 2-sample proportion test, *p*<0.05); nearly 25% of the H>C DCPs (6/25) were involved in the stress response. This relative over-representation of proteins involved in the stress response was mainly driven by the proteomic response of the *Symbiodinium* compartment; only 2 of the 15 coral host DCPs (Fig 2G) were involved in the stress response (not significantly higher than the 6% value of the *P*. *acuta* host transcriptome; 2-sample proportion test, *p*>0.05), and both were documented at higher concentrations in high-temperature gels than in control ones (Fig 2I). In contrast, one-third of the 15 *Symbiodinium* DCPs (Fig 2J) were involved in the stress response, and this was significantly over-represented relative to the *Symbiodinium* transcriptome (4%; 2-sample proportion test, *p*<0.05). Specifically, one and four *Symbiodinium* proteins involved in the stress response were documented at higher concentrations in the control (Fig 2K) and high-temperature (Fig 2L) samples, respectively.

Of the 38 DCPs uncovered at the two-week sampling time, only 4 were associated with an mRNA in which a congruent temperature treatment difference was documented; all were of *Symbiodinium* origin (S1 Table). In all cases, mRNA expression and protein concentration were higher in samples of the high-temperature treatment, and the overall congruency of 10.5% (4/38) was significantly higher than that of a temperature experiment with *S*. *hystrix* [32]: 2% (2-sample proportion test, *p*<0.01). Congruency also differed significantly between compartments (2-sample proportion test, *p*<0.05): 0% for the coral host (0/15 molecules showed a congruent response between mRNA and protein levels.) vs. 27% (4/15) for *Symbiodinium*.

### Four-week sampling time

**Fig 3:**
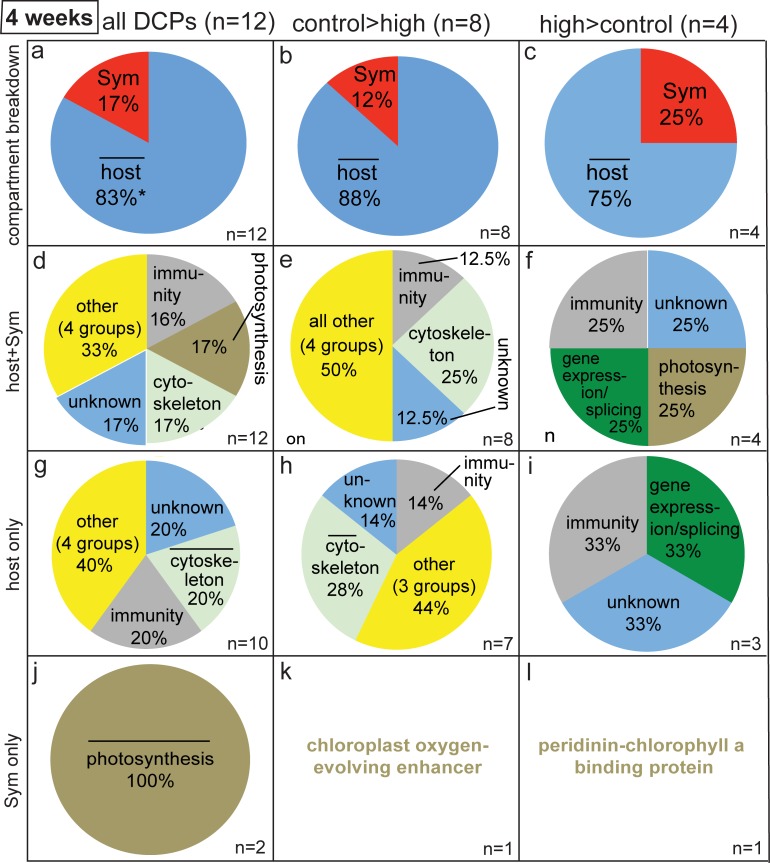
Pie graphs showing breakdown of differentially concentrated proteins (DCPs) between control (C4) and high (H4) temperature samples of the four-week sampling time. Four and four protein spots were found to be more highly concentrated in the C4 (i.e., C>H) and H4 (H>C) proteomes, respectively, and 8 C>H and 4 H>C DCPs were identified from these eight spots. When the host:*Symbiodinium* (Sym) ratio was significantly higher (*z*-test, *p*<0.05) than the *P*. *acuta*:Sym mRNA ratio of 1.9±0.4 (std. dev.), a bar has been inserted over the word “host” in (a-c). Likewise, when a cellular process was over-represented in the proteomes of the host (g-i) and Sym (j-l) relative to the host coral and Sym transcriptomes [29], respectively (2-sample proportion test, *p*<0.05), a bar has been placed over the category name.

At the four-week sampling time, four and four proteins were more highly concentrated by samples of the control (Fig 1C) and high (Fig 1D) temperature treatments, respectively, though none of these were uniquely translated by samples of either (Table 1). From these eight spots, eight C4>H4 and four H4>C4 DCPs were uncovered (Fig 3, Table 2, S2 Table and S5 Table). One DCP identified at the two-week sampling time, host coral pentraxin, was also documented at higher levels in control temperature samples at the four-week sampling time. A hypothetical protein (contig5078) of presumed host origin, in contrast, was more highly concentrated by coral hosts exposed to high temperatures relative to controls at both the two- and four-week sampling times. As such, only 10 of the 12 DCPs identified at the four-week sampling time were unique.

The overall 83/17% host/*Symbiodinium* DCP ratio (i.e., ~5:1) for the 12 DCPs uncovered at the four-week sampling time (Fig 3A) was significantly higher than the coral/*Symbiodinium* ratio of 1 documented across the 38 DCPs of the two-week sampling time (Fig 2A; *X*^2^ test, *p*<0.001), and it was also significantly higher than the 1.9:1 host:*Symbiodinium* mRNA transcript ratio (*z*-test, *p*<0.05). When looking only at the eight proteins that were more highly concentrated by samples of the control temperature treatment at the four-week sampling time (Fig 3B), seven and one were from the coral host and dinoflagellate compartments, respectively, a similar proportional breakdown to that observed for the H>C DCPs (Fig 3C). For a detailed treatise on the functional breakdown of all 12 DCPs of the four-week sampling time (Fig 3D), please see the S1 File, as well as Fig 3E–3L, S2 Table and S5 Table.

### Eight-week sampling time

**Fig 4:**
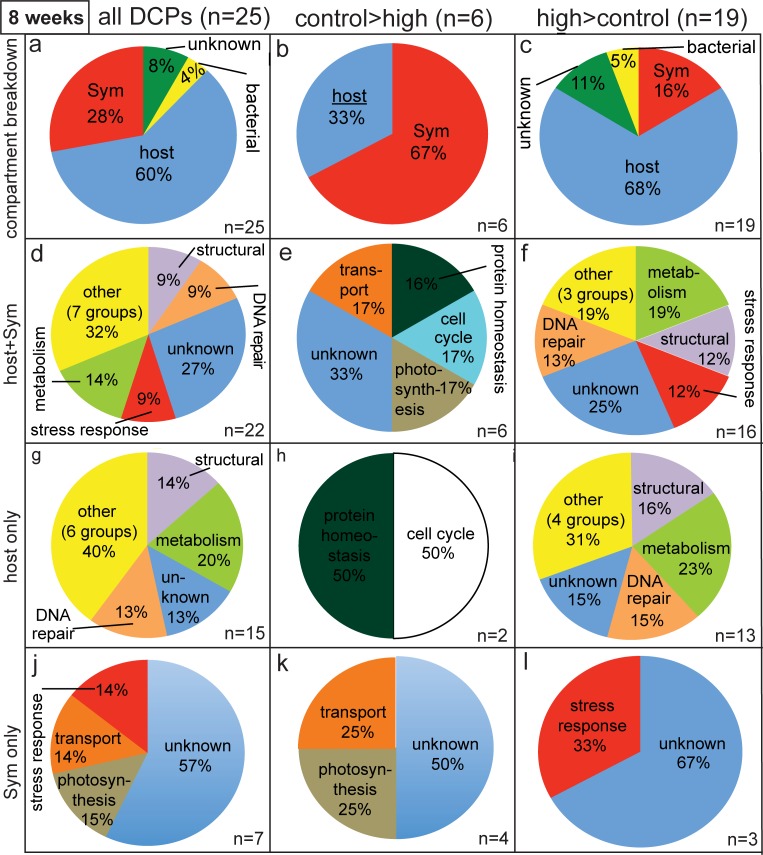
Pie graphs depicting breakdown of differentially concentrated proteins (DCPs) between control (C8) and high (H8) temperature samples of the eight-week sampling time. Two and six spots were more highly concentrated in the C8 (i.e., C>H) and H8 (H>C) proteomes, respectively, and 6 C>H and 19 H>C DCPs were identified from these eight spots. When the host:*Symbiodinium* (Sym) DCP ratio was significantly lower (*z*-test, *p*<0.05) than the *P*. *acuta*: Sym mRNA ratio of 1.9±0.4 (std. dev.), a bar has been inserted under the word “host” in (a-c).

At the eight-week sampling time, two and six protein spots were more highly concentrated by samples of the control (Fig 1E) and high (Fig 1F) temperature treatments, respectively, and one and three of these were uniquely synthesized by samples of the respective treatment (Table 1). From these eight spots, 6 C8>H8 and 19 H8>C8 DCPs were uncovered (Fig 4, Table 2, S3 Table and S6 Table). Two DCPs identified at the two-week sampling time, a *Symbiodinium* protein with both DNAJ and WW domains and a bacterial protein of unknown function, were also documented at higher abundance in high-temperature gels of the eight-week sampling time. A host coral trichohyalin, in contrast, was more highly concentrated by control samples at the four-week sampling time, though instead by high-temperature samples at the eight-week sampling time. As such, of the 25 DCPs identified at the eight-week sampling time, only 22 were unique; 2 were of unknown origin, resulting in 14 unique host and 6 unique *Symbiodinium* DCPs at this sampling time (Table 1). The overall host/Sym DCP ratio was, instead, 15:7 (Fig 4A). For a detailed treatise on the functional and compartmental breakdown of the 25 DCPs uncovered at the eight-week sampling time, please see the S1 File, as well as Fig 4B–4L, S3 Table and S6 Table.

### All sampling times

**Fig 5:**
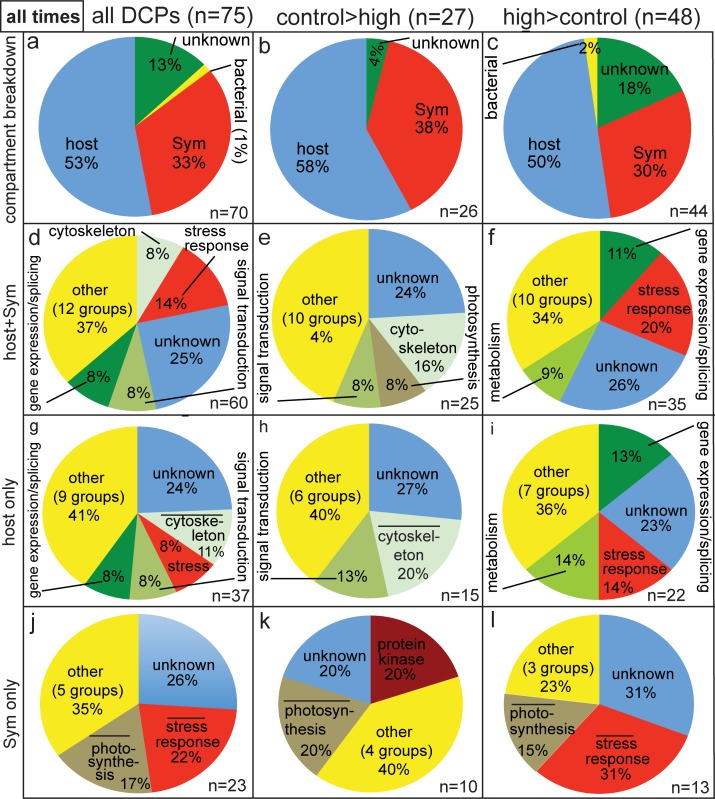
Compartmental and functional breakdown of all differentially concentrated proteins (DCPs). Across the nine, eight, and eight differentially concentrated/uniquely synthesized protein spots removed from representative gels of the two-, four-, and eight-week sampling times, respectively, 75 DCPs were uncovered; upon counting five proteins sequenced at multiple time points only once, 37 and 23 of the 70 unique DCPs were found to be of host coral (*Pocillopora acuta*) and *Symbiodinium* (Sym) origin, respectively. When a cellular process was over-represented in the differentially concentrated proteomes of the host coral (g-i) and Sym (j-l) relative to the host coral and Sym transcriptomes [29], respectively (2-sample proportion test, *p*<0.05), a bar has been inserted above the category name.

**Fig 6:**
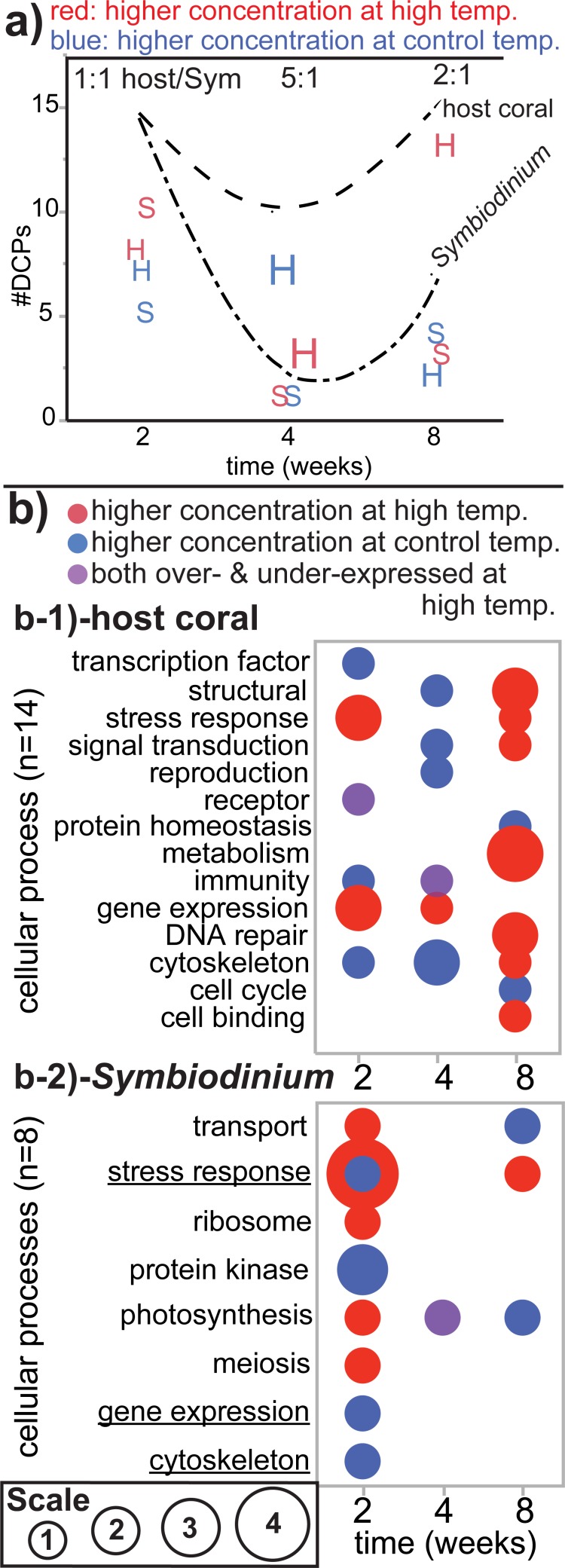
Summary of the dataset. The number of differentially concentrated proteins (DCPs) was plotted over time for both the host coral (“H”) and *Symbiodinium* (“S;” Sym) for proteins documented at higher concentrations at high temperature (red icons) and those over-expressed at the control temperature (blue icons). The sizes of the icons are proportional to the host/Sym DCP ratio at each sampling time (listed at the top of the plot). The upper and lower hatched lines represent the total number of DCPs at each sampling time for the host coral and Sym, respectively. In (b) the sizes of the bubbles are proportional to the number of DCPs involved in each cellular process for both the host coral (b-1) and Sym (b-2). Purple bubbles represent those cellular processes for which one protein was over-expressed at high temperature, whereas another was documented at higher concentrations at the control temperature. This annotation is not used for the Sym “stress response” category at the two-week sampling time (b-2), in which 1 and 4 DCPs were more highly concentrated by the control and high-temperature samples, respectively. Underlined functional categories in b-2 reflect processes that were also temperature-sensitive in the host coral compartment (b-1).

When pooling the data over time (Fig 5), 75 DCPs were sequenced. Since 1) two host coral DCPs were shared between the two- and four-week proteomes, 2) one *Symbiodinium* and one bacterial DCP were sequenced at both the two- and eight-week sampling times, and 3) one host DCP was sequenced at both the four- and eight-week sampling times, 70 unique DCPs were identified (Fig 5A). Of these, 26 and 44 were more highly concentrated by samples of the control (Fig 5B) and high-temperature (Fig 5C) treatments, respectively. One and nine DCPs were of bacterial and unknown origin, respectively, leading to a total of 37 unique host and 23 unique *Symbiodinium* DCPs. This 1.6:1 host/*Symbiodinium* ratio did not differ significantly from the host/*Symbiodinium* transcript of 1.9 (*z*-test, *p*>0.05). The host/*Symbiodinium* DCP ratio did, however, vary over time (*X*^2^ test, *p*<0.05); this was due to the fact that equal numbers of host coral and *Symbiodinium* DCPs were uncovered at the two-week sampling time (1:1; Fig 6A), whereas the four- and eight-week DCP pools featured higher host/*Symbiodinium* DCP ratios (5:1 and 2.1:1, respectively; Fig 6A). *Symbiodinium* were more likely to increase levels of protein abundance in response to experimentally elevated temperatures (n = 10 H>C DCPs) than the host corals in which they resided (n = 8 H>C DCPs) at the earliest sampling time only (*X*^2^ test, *p*<0.05).

The most represented functional categories amongst the 60 host+*Symbiodinium* DCPs were the stress response, cytoskeleton, signal transduction, and gene expression/splicing (Fig 5D and Fig 6B). A more detailed discussion of the compartmental and functional breakdown of these 60 DCPs can be found in the S1 File, Figs 5 and 6, and Table 2. In general, few cellular processes were represented in the proteomes of both compartments. Of the 19 cellular processes featuring at least one DCP, only three were identified in the differentially concentrated proteomes of both host coral and *Symbiodinium*: the stress response, gene expression/splicing, and the cytoskeleton (Fig 6B). Similarly, few cellular processes were represented at more than one sampling time. In the host coral compartment (Fig 6B-1), structural proteins, stress response proteins, signal transduction proteins, and proteins involved in immunity and the cytoskeleton were identified in the differentially concentrated proteomes of multiple sampling times, while for *Symbiodinium* (Fig 6B-2) only three cellular processes were identified at multiple sampling times: transport, the stress response, and photosynthesis. Only proteins involved in the stress response were sequenced (i.e., differentially concentrated) in both cellular compartments of the coral-dinoflagellate endosymbiosis at multiple sampling times (Fig 6B).

## Discussion

### Differential proteomic responses of host corals and their *in hospite Symbiodinium* populations to experimentally elevated temperatures

When attempting to track the responses of multi-compartmental organisms, such as reef-building corals [40–41] or symbiotic forams [42–43], to changes in their environments, it is important to focus not only on the hosts, but also their symbionts [44]. Although *Symbiodinium* comprise ~30–40% of the live biomass of *P*. *acuta*, they contributed an equal number of DCPs as their coral hosts at the two-week sampling time. This suggests that *Symbiodinium* were more strongly affected at the protein level by a two-week elevated temperature exposure than their anthozoan hosts, as was also documented in these same samples at the mRNA level [29]. This does not mean that *Symbiodinium* were more *stressed* than their cnidarian hosts, since they were ultimately found to have acclimated to 30°C [9]. Furthermore, when pooling data across all three sampling times, the host/*Symbiodinium* DCP ratio of 1.6 did not differ significantly from this holobiont’s biomass ratio of ~2:1, meaning that, in contrast to our hypothesis, *Symbiodinium* were only more responsive at the protein-level to elevated temperature exposure than their hosts over a relatively short-term timescale.

Despite the fact that the host coral and *Symbiodinium* exhibited a similar degree of differential protein regulation upon having pooled data across all three sampling times, the cellular processes affected by elevated temperatures tended to differ between the two compartments, and only proteins involved in the stress response were identified at multiple sampling times in both the host coral and *Symbiodinium*. The latter compartment up-regulated stress protein levels to a greater extent than did their coral hosts. Such *Symbiodinium* stress proteins are discussed in more detail below. In contrast, the anthozoan compartment was more likely to differentially concentrate proteins involved in the cytoskeleton, metabolism, and, more generally, processes likely to be involved in osmoadaptation (discussed in more detail below with respect to mechanisms of high-temperature acclimation). Although these data highlight the fact that, in general, different cellular processes were affected by high temperature exposure in each compartment, protein localization and activity data are necessary to more conclusively model the cellular response of the model reef coral *P*. *acuta* and its *in hospite Symbiodinium* populations to elevated temperatures over multi-week timescales.

### Molecular mechanisms of coral acclimation to elevated temperatures

In general, the proteomes of the three sampling times appeared similar on the 2D gels, though the DCPs differed markedly; only 5 of the 75 DCPs (7%) were sequenced at multiple sampling times. Of these five, only host coral pentraxin was down-regulated at high temperature at multiple sampling times. Based on homology searches and studies of vertebrates, pentraxin is likely involved in immunity [45]. A relative decrease in abundance of a single protein putatively involved in immunity at two sampling times does not, in and of itself, suggest that host coral immunity was compromised by high temperature exposure (especially since the corals ultimately acclimated to such elevated temperatures); however, others have instead documented *induction* of immuno-targeted genes in temperature-stressed corals [46], meaning that future works should take a more detailed look at proteins involved in the coral immune system (particularly in corals that are ultimately found to bleach). The decrease in available energy stores coincident with coral bleaching could indeed be predicted to lead to an immuno-compromised state driven by coral malnourishment [47].

In addition to immunity, a number of other cellular processes were affected by elevated temperature exposure. We now use our proteome-scale data to propose two, possibly related cellular processes that may have been involved in the high-temperature acclimation of the experimental corals. We first discuss the role of proteins involved in the stress response and protein homeostasis/turnover. We then turn to the potential importance of osmoregulation in coral acclimation to high temperatures. Regarding the former process, 25 and 40% of the host coral and *Symbiodinium* H>C DCP pools, respectively, comprised proteins involved in the stress response at the two-week sampling time; given that exposure to 30°C was hypothesized to elicit stress, this result is unsurprising. The two host coral H>C stress proteins identified at the two-week sampling time included beta-gamma crystallin and sacsin. Although the former has only ever been hypothesized to be involved in the stress response (as well as calcium binding; [48]), it was found to be down-regulated in *S*. *hystrix* specimens exposed to a variable temperature regime for one week [32]; given its proven temperature sensitivity in multiple experiments featuring different coral models, we recommend that this protein, which is water soluble and frequently documented in the lenses of vertebrate eyes, be functionally characterized in the near future.

With respect to the second of the two host coral proteins up-regulated at high temperatures at the two-week sampling time, sacsin is a co-chaperone of heat shock protein 70 (HSP70), the most well characterized molecular chaperone. Curiously, the expression of neither the host nor *Symbiodinium hsp70* mRNA is affected by short- or long-term high-temperature exposure in Taiwanese corals, presumably because both are synthesized at high levels at all times in preparation for the frequent upwelling events that characterize the reefs of Southern Taiwan [3]. That being said, there is little congruency between gene and protein expression in reef corals or *Symbiodinium* [32]. Therefore, it is possible that the HSP70 proteins with which sacsin associates *were* synthesized at higher levels in the high-temperature samples analyzed herein; they may not have been sequenced due to, for instance, low cellular concentrations (few 70-kDa spots were evident in any of the gels [Fig 1].). Regardless of this discrepancy, a more thorough elucidation of the role of co-chaperones like sacsin and STl1, which was actually down-regulated at high temperatures in *Symbiodinium*, in acclimation to high temperatures represents a worthy endeavor for future study.

This STl1-like protein was the only *Symbiodinium* stress protein down-regulated at high temperatures; in contrast, four *Symbiodinium* stress proteins were up-regulated after two weeks of high-temperature exposure: an uncharacterized protein with DNAJ and WW domains, two peptidylprolyl isomerase D paralogs, and an E3 ubiquitin protein ligase. The former protein has not been characterized and was only hypothesized to be involved in the stress response given its possession of a DNAJ domain, while peptidylprolyl isomerase D proteins are involved in a diverse array of cellular processes aside from the stress response; therefore, it is too speculative at the current time to predict the specific cellular roles of these proteins as they relate to the stress response. It is worth noting that these three proteins were amongst only four (the final being a pentatricopeptide repeat-containing protein) in the entire dataset encoded by mRNAs that also underwent increases in expression in response to exposure to elevated temperatures.

In contrast to the three proteins mentioned in the previous paragraph, E3 ubiquitin protein ligase is both well characterized *and* strongly implicated in the cellular stress response; specifically, this enzyme aids in the process by which an E2 ubiquitin-conjugating enzyme transfers a ubiquitin tag to a functionally compromised protein slated to be degraded by the proteasome [49]. As such, this protein is involved in protein homeostasis and turnover, a process we hypothesized previously [29] (reiterated in the Introduction) to be important in coral high-temperature acclimation. The elevated concentrations of E3 ubiquitin protein ligase and HSP70 co-chaperones in both compartments of *P*. *acuta-Symbiodinium* endosymbioses exposed to high temperatures for two weeks suggests that protein turnover in these same samples was relatively high. This may be indicative of incomplete acclimation or even baseline levels of stress (e.g., due to photoinhibition [50], reactive oxygen species production [51], or osmotic pressure decreases [discussed in more detail below]). However, as corals survived for nine months at 30°C, this protein degradation/folding-mediated stress response was clearly able to restore homeostasis to the extent that sufficient energy could be shuttled to growth-related processes (growth was similar between treatments [9].). A look at the additional cellular processes affected by elevated temperature exposure may further elucidate how such high-temperature acclimation occurred.

Maintenance of osmotic pressure is the most energetically expensive task a cell undertakes [52], and coral-*Symbiodinium* osmoregulation has been hypothesized to be impacted by elevated temperature exposure [53]. This hypothesis stems from the notion that cessation of osmolyte flux from *Symbiodinium* to the host coral as a result of high temperature-induced photoinhibition [50] could result in a drop in the osmotic pressure of the host gastrodermal cell. Indeed, *Symbiodinium* photosynthesis may have been influenced by high temperature exposure herein given the number of differentially concentrated photosynthesis-associated proteins uncovered (e.g., ribulose-1,5-bisphosphate carboxylase/oxygenase, fucoxanthin-chlorophyll a-c binding protein F, chloroplast oxygen-evolving enhancer, and peridinin-chlorophyll A binding protein). Such a decrease in osmotic pressure could lead to a consequent collapse of the coral plasma membrane upon the *Symbiodinium* cell [54]. This would necessitate a regulatory volume increase, in which, amongst a plethora of processes, the cytoskeleton must be rebuilt [55]. This may explain why a number of actins and other cytoskeleton proteins (e.g., spectrin, trichohyalin, and stabilizer of axonemal microtubules 2-like) differed in concentration between temperature treatments in the coral host in particular; such has also been documented at the mRNA-level in thermally challenged corals [56]. Re-establishment of osmotic pressure, then, may have allowed for these corals to have maintained cellular homeostasis at the experimentally elevated temperature of 30°C, though gastrodermal cell-specific proteomic approaches (*sensu* [10]) are needed to substantiate this hypothetical role of osmoadaptation, as well as the protein turnover-related processes described above, in coral acclimation to elevated temperatures. It is worth noting that, since *Symbiodinium* possess rigid, durable cell walls, osmotic stress is not hypothesized to affect their cell volume [53].

### Low congruency between gene expression and protein concentration

At the two-week sampling time, only 4 of the 38 DCPs were associated with an mRNA whose expression also differed significantly between treatments. All four of these molecules were expressed at higher levels by *Symbiodinium* within corals exposed to elevated temperatures, and *Symbiodinium* were significantly more likely to be characterized by congruency between mRNA expression and protein concentration (27%) than the coral hosts in which they resided (0%). This 27% congruency is significantly higher than that of another study; not a single *Symbiodinium* molecule showed a similar temperature-related difference at the mRNA and protein levels in cells housed within the gastroderms of *S*. *hystrix* [32].

The immense sizes of the *P*. *acuta* and *Symbiodinium* transcriptomes (>100,000 contigs each) may account for, in part, the low degree of congruency between mRNA expression and protein abundance; for many of the genes of both *P*. *acuta* and *Symbiodinium*, multiple splice variants exist [29]. Given that the actual translation of these mRNAs was not observed, it cannot be known which splice variants were actually translated into proteins. Like Mascot (Matrix Sciences), MS-SCAN/MS-GF+ makes guesses about protein identity based on querying translated transcriptomes; no coral proteomes have been sequenced at present, and only a partial sea anemone proteome (~3,000 proteins from *Aiptasia* sp.) currently exists [57]. Simply because MS-SCAN yields a top hit for a particular peptide sequence does not mean that the “correct” splice variant was targeted; the peptides sequenced by MS are typically short (10–15 AA) and so may align to multiple splice variants. It is possible that improvements in protein sequencing will lead to longer AA “reads,” in which case there will be greater confidence in having isolated the splice variant encoding the protein of interest [58]. This will allow us to know whether the congruency between mRNA and protein concentration is indeed only 0–27% and 0–2% for *Symbiodinium* and pocilloporid corals, respectively. Until such can be verified, it is unadvised to use mRNA-level data alone to make inferences about the cellular behavior of reef corals, especially with respect to their ability to acclimate to environmental change (*sensu* [59–60]).

## Supporting information

S1 File Supplemental methods and results. (DOCX)

S1 Table Proteins whose concentrations differed between temperature treatments at the two-week sampling time. Underlined spots represent uniquely synthesized proteins (see Fig 1 and Table 1.). Congruent and marginally congruent results between gene expression and protein concentration are highlighted in green and purple, respectively. Proteins involved in the stress response are highlighted in red. Proteins highlighted in blue and yellow were documented at different concentrations between treatments at the four- and eight-week sampling times, respectively (Table 2). Please see the S4 Table for peptide sequences. “C” and “H” in the “Spot” column correspond to spots removed from the control and high-temperature treatment gels, respectively. In the “mRNA effect” column, “C2” and “H2” correspond to control and high-temperature samples at the two-week sampling time, respectively, while “C36” and “H36” correspond to control and high-temperature samples at the 36-week sampling time, respectively. AA = amino acid. kDa = kilodalton. MW = molecular weight. NS = not significant (neither temperature nor time effect in the repeated measures ANOVA [*p*>0.05]). pI = isoelectric point. Sym = *Symbiodinium*.
(DOCX)

S2 Table Proteins whose concentrations differed between temperature treatments at the four-week sampling time. Proteins highlighted in blue and grey also differed in concentration between treatments at the two- and eight-week sampling times, respectively (Table 2). Please see the S5 Table for hypothetical functions and peptide sequences. “C” and “H” in the “Spot” column correspond to spots removed from the control and high-temperature treatment gels, respectively. AA = amino acids. DCP = differentially concentrated protein. kDa = kilodalton. MW = molecular weight. pI = isoelectric point. Sym = *Symbiodinium*.
(DOCX)

S3 Table Proteins whose concentrations differed between temperature treatments at the eight-week sampling time. Underlined spots represent uniquely synthesized proteins (see Fig 1 and Table 1.). Proteins highlighted in yellow and grey also differed in concentration between treatments at the two- and four-week sampling times, respectively (Table 2), and those highlighted in red were hypothesized to be involved in the stress response. Please see the S6 Table for hypothetical functions and peptide sequences. “C” and “H” in the “Spot” column correspond to spots removed from the control and high-temperature treatment gels, respectively. AA = amino acids. kDa = kilodalton. MW = molecular weight. pI = isoelectric point. Sym = *Symbiodinium*.
(DOCX)

S4 Table Peptide sequences for proteins whose concentrations differed between temperature treatments at the two-week sampling time. The 13 and 25 unique proteins whose concentrations were higher in samples of the control (C) and high temperature (H) treatments, respectively, at the two-week sampling time were included. Additional details of the sequenced proteins can be found in the S1 Table. Although multiple missed cleavages exist in certain peptide sequences (more than the two allowed by the MS-GF+ script [described in the supplemental methods section of the S1 file]), these peptides were nevertheless included provided that they were either 1) 15 or more amino acids (AA) in length or 2) paired with one or more additional peptides that mapped to the same reference protein (whose collective length summed to 15 or more AA). The compartment of origin has been mentioned next to the spot number when it could be determined. Of the 38 proteins identified by BLAST analysis of the top hit contig (mRNA) derived from MS-SCAN analysis of the *Pocillopora acuta-Symbiodinium* (“Sym”) transcriptome (see the S1 Table for contig accession numbers.), the identities of 12 were further verified by directly BLASTing the MS-SCAN-derived peptide sequences against the *Stylophora pistillata* (n = 8) and *Symbiodinium* (clade B1) genomes (n = 4).
(DOCX)

S5 Table Peptide sequences for proteins whose concentrations differed between temperature treatments at the four-week sampling time. The eight and four proteins whose concentrations were higher in samples of the control (C) and high temperature (H) treatments, respectively, at the four-week sampling time were included. Additional details of the sequenced proteins can be found in the S2 Table. Although multiple missed cleavages exist in certain peptide sequences (more than the two allowed by the MS-GF+ script [desribed in the supplemental methods outlined in the S1 file]), these peptides were nevertheless included provided that they were either 1) 15 or more amino acids (AA) in length or 2) paired with one or more additional peptides that mapped to the same reference protein (whose collective length summed to 15 or more AA). No unique, differentially concentrated proteins were identified from spots H5 and H6. The compartment of origin has been included next to the spot number. Of the 12 proteins identified by BLAST analysis of the top hit contig derived from MS-SCAN analysis of the *Pocillopora acuta-Symbiodinium* (“Sym”) transcriptome (see the S2 Table for contig accession numbers.), the identies of half were further verified by directly BLASTing the MS-SCAN-derived peptide sequences against the *Stylophora pistillata* (n = 4) and *Symbiodinium* (clade B1) genomes (n = 2).
(DOCX)

S6 Table Peptide sequences for proteins whose concentrations differed between temperature treatments at the eight-week sampling time. The 6 and 19 proteins whose concentrations were higher in samples of the control (C) and high (H) temperature treatments, respectively, at the eight-week sampling time were included. Additional details of the sequenced proteins can be found in the S3 Table. Although multiple missed cleavages exist in certain peptide sequences (more than the two allowed by the MS-GF+ script [described in the supplemental methods outlined in the S1 file]), these peptides were nevertheless included provided that they were either 1) 15 or more amino acids (AA) in length or 2) paired with one or more additional peptides that mapped to the same reference protein (whose collective length summed to 15 or more AA). The lysine- and arginine-rich nature of the *Pocillopora acuta* and *Symbiodinium* (“Sym”) proteomes of this study, as well as another carried out with the con-familial pocilloporid coral *Seriatopora hystrix* [32], suggests that alternative protein digest enzymes may serve better for future proteomic analyses of pocilloporid corals. The compartment of origin has been included next to the spot number when it could be determined. Of the 25 proteins identified by BLAST analysis of the top hit contig derived from MS-SCAN analysis of the *P*. *acuta-Symbiodinium* transcriptome (see the S3 Table for contig accession numbers.), the identities of 11 were further verified by directly BLASTing the MS-SCAN-derived peptide sequences against the *Stylophora pistillata* (n = 8) and *Symbiodinium* (clade B1) genomes (n = 3). bact = bacterial.
(DOC)
