# Supplementary material for: The proteomic response of the reef coral Pocillopora acuta to experimentally elevated temperatures
Source: PLoS One. 2018 Jan 31;13(1):e0192001. doi: 10.1371/journal.pone.0192001 (PMC5792016; doi:10.1371/journal.pone.0192001)
Supplement: S1 File — (DOCX) [file pone.0192001.s001.docx]

**S1 file-supplemental methods and results**

**Supplemental methods**

Protein extraction

The proteins (three from each of two treatments at each of three sampling times; n=18) were originally dissolved in SDS-PAGE sample buffer, which is unsuitable for two-dimensional (2D) gel electrophoresis. Therefore, they were precipitated in acetone at -80°C overnight. After centrifugation at 12,000 *xg* for 10 min at 4°C, proteins pellets were washed thrice with a solution comprised of 0.3 M guanidine HCl in 95% ethanol (with 2.5% glycerol). They were then washed once with a solution comprised of 95% ethanol with 2.5% glycerol before drying on the benchtop for 10 min. Protein pellets were dissolved in 0.2 ml of urea rehydration buffer (9.5 M urea, 2% CHAPS, 0.5% carrier ampholytes, and 65 mM dithiothreitol), vortexed vigorously for 1 hr, spun at 12,000 *xg* for 10 min, and the supernatants were transferred to a new tube. Then, 20 μl were quantified with the 2D-Quant™ kit (Amersham Biosciences) according to the manufacturer’s recommendations.

2D gel electrophoresis

Isoelectric focusing (IEF) was used for the first dimension, and the Ettan IPGphor IEF system (Amersham Biosciences) was utilized. Along the center of the bottom of the IEF strip holder, proteins (150 μg/sample; 125 μl) were loaded evenly from left to right, ensuring that there were no air bubbles. The protective membrane was removed from the IEF strip (pH 4-7, 7 cm, Amersham Biosciences), which was then placed gel side down into the strip holder. Then, 200 μl of dry strip cover fluid were coated atop the strip, and the lid was placed over the strip holder. The two strip holder units (one for each of the two co-run samples) were placed in the Ettan IPGphor IEF electrophoresis chamber (Amersham Biosciences), and the following program was run at 20°C: 50 V for 12 hr (rehydration), 300 V for 60 V-hr along a gradient, 600 V for 120 V- hr along a gradient, 1000 V for 500 V-hr along a gradient, 2000 V for 1000 V-hr along a gradient, 5000 V for 6000 V-hr, and 50 V for 10 hr.

Upon completion of IEF, Whatman chromatography paper was cut to a 1 x 0.5 cm size and overlaid with 5 μl of protein marker (Fermentas PageRuler™ prestained protein ladder, Life Technologies). Then, ~1 ml of 1% agarose was coated onto a smooth sheet of plastic wrap, and the chromatography paper was placed over the agarose. An additional 1 ml of 1% agarose was then overlaid on the chromatography paper. After the agarose solidified, the chromatography paper was removed from the agarose to where a 1 mm distance was maintained around the paper. Meanwhile, the IEF strips were immersed in equilibration buffer (6 M urea, 2% SDS, 30% glycerol, 50 mM Tris-HCl [pH 8.8], 0.002% bromophenol blue, and 1% DTT) at room temperature (RT) for 15 min. Then, strips were transferred to the same buffer, except with 1% iodoacetamide (IAA) instead of DTT, for 15 min at RT, washed with SDS-PAGE running buffer to remove residual IAA, and placed on top of a 5-14% stacking-separating Tris-glycine SDS-PAGE gel. Electrophoresis was conducted on ice at 70 V for ~30 min and 120 V for 1-2 hr in a Mini-PROTEAN® Tetra cell (BioRad), with two samples (one from the control treatment and one from the high-temperature treatment) run at the same time. In total, 18 2D gels were run, though only two gels were run at any given time (nine days were required to run all 18 gels.).

Each gel was fixed in 50% methanol and 7% acetic acid for 30 min after removing the stacking gel. Then, the gels were stained with SYPRO® Ruby (Life Technologies) on a shaker table in the dark overnight. The gels were then destained in 10% methanol and 7% acetic acid for 30 min and imaged with a Typhoon Trio™ scanner (GE Healthcare) at 312 nm (aperture=2.8, exposure time=2.4 s). Differentially concentrated proteins (DCPs) were identified and removed from the representative gels as described in the main text.

Preparation of samples for mass spectrometry

Protein + gel slabs were washed with 50% acetonitrile in 25 mM ammonium bicarbonate (pH 8.5). Then, they were incubated in 100 μl of the same acetonitrile solution for 15 min and spun at 10,000 *xg* for 1 min. The supernatant was removed and replaced with 100 μl of 100% acetonitrile, and the samples were incubated for 5 min. The samples were spun again as above, and the supernatant was removed. The gel bits were allowed to dry for 5 min before incubation with 30 μl trypsin (a 2 μg aliquot that had been re-suspended in 1 ml of water and 1 ml of 50 mM ammonium bicarbonate) at 37°C overnight. The next day, samples were centrifuged at 10,000 *xg* for 1 min, and the supernatant was transferred to a new microcentrifuge tube. Then, 50 μl of 50% acetonitrile and 5% trifluoroacetic acid (TFA) were added to the remaining samples, which were sonicated 10 times (10 s each time). Samples were centrifuged again at 10,000 *xg* for 1 min, and the supernatant was combined with the supernatant from the first spin. Another round of 50% acetonitrile/5% TFA incubation followed by sonication/spinning/supernatant collection was conducted, and the third supernatant was combined with the previous two. The supernatant was dried for 1-2 hr prior to shipping to the mass spectrometry (MS) facility at Kaohsiung Medical University’s Center for Research Resources and Development’s Core Proteomics Facility. MS was carried out as described in the main text.

MS-SCAN

The MS-SCAN is based on the script “MS-GF+” written by Kim and Pevner [1]. Their original script can be found on “github” and <http://omics.pnl.gov/software/ms-gf>. MS-GF+ has been shown to generate more peptide-spectral matches (PSM) than Mascot+Percolator (Matrix Sciences) at the same run time [1], and, unlike Mascot, it is free. All default parameters of MS-GF+ were used, and this included a parent (precursor) mass tolerance of 20 parts per million (ppm; equivalent to 2.5 Da), a minimum peptide length of 6 amino acids (AA; see additional minimum length requirements in the main text), and a maximum peptide length of 40 AA. Up to two missed cleavages were allowed, though because impartial trypsin digestion has not been shown to cause quantitative bias in MS-based proteomic applications [2], impartially digested peptides were generally maintained in the analysis provided that they fulfilled the additional inclusion criteria outlined in the main text. Additional default conditions used in the analysis can be found on <http://omics.pnl.gov/software/ms-gf>. Only “rank 1” proteins were considered, and the PSM-level false discovery rate (FDR) was set to 1%; this calculation was based on *q*-values provided by MS+GF+, and details on such *q*-value calculations and filtering can be found in a published work [3].

Data analysis

There was an interest in knowing whether those proteins found to be differentially concentrated between temperature treatments were associated with mRNAs that showed similar temperature-related differences (i.e., “congruency”). Since 2D+MS data are semi-quantitative, whereas RNA-Seq data are quantitative, a correlation analysis was not possible. Instead, repeated-measures ANOVAs were used to determine the effects of temperature (control vs. high), time (2 vs. 36 weeks), and their interaction on the mRNA expression data. When significant interaction effects were documented (*p*<0.05), Tukey’s honestly significant difference (HSD) tests were performed between temperature treatments at the two-week sampling time: control-time 2-week (C2) vs. high-temperature-time 2-week (H2). When a gene was associated with a protein that showed the same treatment-related difference at the two-week sampling time, “congruency” between mRNA expression and protein concentration was said to have occurred. For instance, if mRNA expression of a gene was significantly higher in samples of the C2 treatment relative to the H2 treatment, and the respective DCP was sequenced from the final, representative C2 2D gel, then the mRNA and protein-level findings would be said to be congruent. Two-sample proportion tests were used to determine whether congruency differed significantly across compartments, as well as between this study and one featuring *S. hystrix* [4].

Supplemental results

Four-week sampling time

Please see the main text for a discussion on the compartmental breakdown of the 12 DCPs uncovered at the four-week sampling time (Fig 3, Table 2, S2 table, and S5 table). The most represented functional categories were immunity (host coral only), photosynthesis (*Symbiodinium* only), and cytoskeleton (both compartments). None of these categories were over-represented relative to the two-week DCP pools (see main text; *X*^2^ tests, *p*>0.05). When looking only at the eight C>H DCPs, cytoskeleton was the most represented functional category (Fig 3e), and the four H>C DCPs (Fig 3f) were involved in the following three cellular processes: gene expression/splicing, photosynthesis, and immunity.

Of the 10 host coral time=four-week DCPs (Fig 3g), the most represented functional categories were cytoskeleton and immunity, and such was also the case for the seven C>H host proteins (Fig 3h). In contrast, immunity and gene expression/splicing were the two most represented cellular processes for the three host H>C proteins (Fig 3i). There was a higher proportion of cytoskeleton proteins than in the transcriptome (Fig 3g); 4% of the transcriptome is comprised of cytoskeleton genes [5], whereas 20% of the differentially concentrated host coral proteome was comprised of proteins associated with the cytoskeleton (2-sample proportion test, *p*<0.05). Cytoskeleton proteins were also over-represented relative to the transcriptome for the C>H host coral differentially concentrated proteome (Fig 3h; 2-sample proportion test, *p*<0.05). Only two *Symbiodinium* DCPs were uncovered at the four-week sampling time (Fig 3j), and both were involved in photosynthesis (Fig 3k-l). Photosynthesis-associated proteins were over-represented in the four-week *Symbiodinium* proteomes relative to the two-week *Symbiodinium* proteomes (*X*^2^ test, *p*<0.05), and they were also over-represented relative to their proportion in the *Symbiodinium* transcriptome (2-sample proportion test, *p*<0.05).

Eight-week sampling time

The 60/28% host/*Symbiodinium* DCP ratio for the 25 DCPs uncovered at the eight-week sampling time (~2; Fig 4a) was significantly higher than the coral/*Symbiodinium* DCP ratio of 1 documented at the two-week sampling time (Fig 2a; *X*^2^ test, *p*<0.001), though it did not differ significantly from the host/*Symbiodinium* mRNA transcript ratio of 1.9 (*z*-test, *p*>0.05). When looking only at the six DCPs over-expressed by samples of the control treatment at the eight-week sampling time (Fig 4b), two and four were from the coral host and dinoflagellate compartments, respectively; this differed markedly from the H>C DCP pool (Fig 4c), in which the majority of the 19 DCPs were of host origin (Table 2, S3 table, and S6 table). Regarding the functional breakdown of all 22 host+*Symbiodinium* DCPs (Fig 4d), the most represented cellular processes were metabolism, the stress response, DNA repair, and cell structure. When looking only at the six C>H DCPs, photosynthesis, transport, protein homeostasis, and cell cycle were the most represented processes (Fig 4e). Finally, a wide variety of cellular pathways were represented across the 16 H>C DCPs (Fig 4f), including the stress response, metabolism, and DNA repair.

Of the 15 host coral eight-week DCPs (Fig 4g), the most represented cellular processes were metabolism, DNA repair, and cell structure; such was also the case for the 13 H>C host proteins (Fig 4i). In contrast, protein homeostasis and cell cycle were the lone pathways represented across the two host C>H DCPs (Fig 4h). Only seven *Symbiodinium* DCPs were uncovered at the eight-week sampling time (Fig 4j), and the majority could not be assigned an identity. That being said, half of the four *Symbiodinium* DCPs uncovered in the two C>H spots were involved in photosynthesis and transport, and one of the three H>C DCPs was involved in the stress response.

All sampling times

In total 60 unique host+*Symbiodinium* proteins were differentially concentrated between experimental temperatures across the two-, four-, and eight-week sampling times (Fig 5). Please see the main text for a more thorough discussion of the compartmental breakdown of these proteins (Fig 5a-d and Table 2). For the 25 C>H DCPs across both compartments (Fig 5e), signal transduction, cytoskeleton, and photosynthesis were the most represented cellular processes. The stress response, metabolism, and gene expression/splicing were the most represented amongst the 35 H>C DCPs across both compartments (Fig 5f). Of the 37 unique host coral DCPs across all three sampling times (Fig 5g), cytoskeleton was the most represented process, as was also the case for the 15 C>H host coral DCPs (Fig 5h). In contrast, the stress response, metabolism, and gene expression/splicing were the most represented categories in the 22 H>C host DCP pool (Fig 5i). Host cytoskeleton proteins were significantly over-represented relative to the host coral transcriptome for both the total host DCP pool (Fig 5g) and the C>H host DCP pool (Fig 5h; 2-sample proportion tests, *p*<0.05).

Proteins involved in the stress response and photosynthesis were relatively more represented in the DCP pools of *Symbiodinium* (Fig 5j); 20% of the 10 C>H DCPs were involved in photosynthesis (Fig 5k), and 31% of the 13 H>C *Symbiodinium* DCPs were involved in the stress response (Fig 5l). Both such categories were over-represented relative to their respective proportions in the *Symbiodinium* transcriptome (2-sample proportion tests, *p*<0.0001), in which 0.6 and 4% of the genes encode proteins involved in photosynthesis and the stress response, respectively [5].

Supplemental references

1. Kim S, Pevzner PA. MS-GF+ makes progress towards a universal database search tool for proteomics. Nature Commun*.* 2014;5: 5277.
2. Chiva C, Ortega M, Sabido E. Influence of the digestion technique, protease, and missed cleavage peptides in protein quantitation. J Proteome Res*.* 2014;13(9): 3979-3986.
3. Rudnick PA, Markey SP, Roth J, Mirokhin Y, Yan X, Tchekhovskoi DV, et al. A description of the clinical proteomic tumor analysis consortium (CPTAC) common data analysis pipeline. J Proteome Res. 2016;15(3): 1023-1032.
4. Mayfield AB, Wang YB, Chen CS, Chen SH, Lin CY. Dual-compartmental transcriptomic+proteomic analysis of a marine endosymbiosis exposed to environmental change. Mol Ecol*.* 2016;25: 5944-5958.
5. Mayfield AB, Wang YB, Chen CS, Chen SH, Lin CY. Compartment-specific transcriptomics in a reef-building coral exposed to elevated temperatures. Mol Ecol. 2014;23: 5816-5830.
