# Supplementary material for: The proteomic response of the reef coral Pocillopora acuta to experimentally elevated temperatures
Source: PLoS One. 2018 Jan 31;13(1):e0192001. doi: 10.1371/journal.pone.0192001 (PMC5792016; doi:10.1371/journal.pone.0192001)
Supplement: S1 Table — Underlined spots represent uniquely synthesized proteins (see Fig 1 and Table 1.). Congruent and marginally congruent results between gene expression and protein concentration are highlighted in green and purple, respectively. Proteins involved in the stress response are highlighted in red. Proteins highlighted in blue and yellow were documented at different concentrations between treatments at the four- and eight-week sampling times, respectively (Table 2). Please see the S4 Table for peptide sequences. “C” and “H” in the “Spot” column correspond to spots removed from the control and high-temperature treatment gels, respectively. In the “mRNA effect” column, “C2” and “H2” correspond to control and high-temperature samples at the two-week sampling time, respectively, while “C36” and “H36” correspond to control and high-temperature samples at the 36-week sampling time, respectively. AA = amino acid. kDa = kilodalton. MW = molecular weight. NS = not significant (neither temperature nor time effect in the repeated measures ANOVA [p>0.05]). pI = isoelectric point. Sym = Symbiodinium. (DOCX) [file pone.0192001.s002.docx]

**S1 table. Proteins whose concentrations differed between temperature treatments at the two-week sampling time.** Underlined spots represent uniquely synthesized proteins (see Fig 1 and Table 1.). Congruent and marginally congruent results between gene expression and protein concentration are highlighted in green and purple, respectively, in the “mRNA effect” column. Proteins involved in the stress response are highlighted in red. Proteins highlighted in blue and yellow were documented at different concentrations between treatments at the four- and eight-week sampling times, respectively (Table 2). Please see the S4 table for peptide sequences. When two accession numbers have been included for the same protein, the top and bottom correspond to the top hit acquired upon BLASTing the sequence of the mRNA encoding the sequenced peptide and the top hit acquired upon BLASTing the peptide sequence itself, respectively. “C” and “H” in the “Spot” column correspond to spots removed from the control and high temperature treatment gels, respectively. In the “mRNA effect” column, “C2” and “H2” correspond to control and high-temperature samples at the two-week sampling time, respectively, while “C36” and “H36” correspond to control and high-temperature samples at the 36-week sampling time, respectively. AA=amino acid. kDa=kilodalton. MW=molecular weight. NS=not significant (neither temperature nor time effect in the repeated measures ANOVA [*p*>0.05]). pI=isoelectric point. Sym=*Symbiodinium*.

| **Spot** | **Protein** | | **NCBI accession of top hit(s)** | **Top hit taxon** | **# Unique peptides** | **#AA se-quenced** | **% Co-verage** | **Compart-ment** | ***Pocillopora acuta* transcriptome contig** | **mRNA effect** |
| --- | --- | --- | --- | --- | --- | --- | --- | --- | --- | --- |
| **Spot C1: higher concentration in control samples** (n=5 [2 were discarded]). Spot pI=4.3. MW=34 kDa. | | | | | | | | | | |
| C1-2 | pentraxin | | XP_001621255 | anemone | 4 | 69 | 29 | host | comp40757_c0_seq1 | H36>C36 |
| C1 | transcription factor death-induced obliterator-1 | | XP_022798202  XP_015774430 | coral | 2 | 32 | 2 | host | comp124214_c0_seq3 | NS |
| C1 | protein kinase UbiB | | OLQ11043 | dinoflagellate | 2 | 47 | 7 | Sym | comp233055_c0_seq1 | 2>36 |
| C1 | STI1-like protein | | OLP87961 | dinoflagellate | 2 | 41 | 7 | Sym | comp94422_c0_seq1 | H>C, 2>36 |
| C1 | myosin-6 | | OLP95342 | dinoflagellate | 2 | 47 | 4 | Sym | comp82970_c0_seq1 | H>C, 2>36 |
| **Spot C2: higher concentration in control samples** (n=3 [3 were discarded and 1 was repeated]). Spot pI=4.6. MW=19 kDa. | | | | | | | | | | |
| C2 | WSC domain | | XP_015768566 | coral | 2 | 56 | 17 | host | comp106562_c0_seq2 | NS |
| C2 | cadherin EGF LAG seven-pass G-type receptor 2 | | XP_015760091PFX24105 | coral | 2 | 57 | 3 | host | comp123702_c0_seq2 | 36>2 |
| C2 | pre-mRNA-splicing factor SLU7-A | | OLP92736 | dinoflagellate | 2 | 27 | 4 | Sym | comp93864_c0_seq1 | H>C, 2>36 |
| **Spot C3: higher concentration in control samples** (n=5 [3 were discarded]). Spot pI=6.2. MW=24 kDa. | | | | | | | | | | |
| C3 | hypothetical protein | | KXJ07568 | anemone | 3 | 51 | 14 | host | Contig3115 | NS |
| C3 | Pao retrotransposon peptidase | | XP_015763616 | coral | 2 | 42 | 2 | host | comp122074_c1_seq2 | NS |
| C3 | spectrin alpha chain | | XP_015751065  XP_022807090 | coral | 3 | 71 | 2 | host | comp124294_c0_seq2 | H36>H2 |
| C3 | adenylate kinase | | CEM16623 | alveolate | 2 | 37 | 2 | Sym | comp88313_c0_seq1 | NS |
| C3 | hypothetical protein | | no hit | no hit | 2 | 22 | 20 | unknown | Contig10927 | NS |
| **Spot H1: higher concentration in high temperature samples** (n=5 [2 were discarded and 1 was repeated]). Spot pI=4.6. MW=24 kDa. | | | | | | | | | | |
| H1 | hypothetical protein | | KXJ05512 | anemone | 2 | 20 | 7 | host | Contig5078 | NS |
| H1, H3 | low-density lipoprotein receptor-related protein 4 | | KXJ24053 &  XP_022802515 | anemone & coral | 2 | 45 | 5 | host | Contig3789 | 36>2,  H>C (*p*=0.06) |
| H1 | serine/arginine repetitive matrix protein 1^a^ | | KXJ23774 XP_022796060 | anemone & coral | 2 | 34 | 4 | host | Contig2169 | H36>C2, 36>2 |
| H1 | Rec10/Red1 | | OLP96989 | dinoflagellate | 2 | 32 | 13 | Sym | comp188028_c0_seq1 | H>C (*p*=0.06) |
| H1 | VWF domain-containing protein | | WP_008002149 | bacteria | 2 | 31 | 13 | bacteria^b^ | Contig14091 | H36>C2, 36>2 |
| **Spot H2: higher concentration in high temperature samples** (n=5 [3 were discarded and 1 was repeated]). Spot pI=6.2. MW=73 kDa. | | | | | | | | | | |
| H2 | hypothetical protein | | XP_015769587  XP_022782362 | coral | 2 | 62 | 14 | host | comp119738_c0_seq7 | NS |
| H2 | histone-lysine N-methyltransferase SETD1B-like | | XP_015763737 XP_022801012 | coral | 2 | 29 | 1 | host | Contig6306 | 36>2 |
| H2 | ribulose-1,5-bisphosphate carboxylase/oxygenase^c^ | | AF298221_1 | dinoflagellate | 5 | 76 | 31 | Sym | Contig12235^d^ | 2>36, H>C (no *post-hoc* differences) |
| H2-3 | protein w/ DNAJ and WW domains | | OLP82285 | Sym | 2 | 18 | 4 | Sym | comp109287_c2_seq1 | H2>all others |
| H2 | hypothetical protein | | No hit | no hit | 2 | 37 | 53 | unknown | comp596053_c0_seq1 | NS |
| **Spot H3: only translated by high temperature samples** (n=4 [4 were discarded and 2 were repeated]). Spot pI=6.0. MW=29 kDa. | | | | | | | | | | |
| H3 | golgin subfamily B member 1 | XP_015764529.1  XP_022806499 | | coral | 2 | 40 | 1 | host | Contig7281 | 36>2 |
| H3 | peptidylprolyl isomerase D | OLQ10279 | | dinoflagellate | 2 | 18 | 3 | Sym | comp41252_c0_seq1 | H2>C2, H>C, 2>36 |
| H3 | hypothetical protein | No hit | | no hit | 2 | 30 | 38 | unknown | comp745911_c0_seq1 | NS |
| H3 | hypothetical protein | No hit | | no hit | 2 | 49 | 16 | unknown | comp97361_c3_seq1 | 2>36 |
| **Spot H4: only translated by high temperature samples** (n=3 [3 were discarded and 2 were repeated]). Spot pI=6.1. MW=29 kDa. | | | | | | | | | | |
| H4 | E3 ubiquitin protein ligase | XP_013904630 | | algae | 2 | 39 | 18 | Sym | comp258540_c0_seq1 | NS |
| H4 | hypothetical protein | No hit | | no hit | 2 | 38 | 31 | unknown | mira_454_illumina_rep_c11366 | NS |
| H4 | hypothetical protein | No hit | | no hit | 3 | 24 | 14 | unknown | comp109527_c0_seq1 | NS |
| **Spot H5: only translated by high temperature samples** (n=3 [2 were discarded]). Spot pI=6.5. MW=28 kDa. | | | | | | | | |  |  |
| H5-6 | beta-gamma crystallin | ABV24977 | | coral | 3 | 62 | 29 | host | Contig13480 | NS |
| H5 | sacsin | XP_015762902.1  PFX28335 | | coral | 2 | 50 | 1 | host | Contig7988 | NS |
| H5 | hypothetical protein | No hit | | no hit | 2 | 24 | 33 | unknown | comp74379_c3_seq1 | NS |
| **Spot H6: only translated by high temperature samples** (n=5 [3 were discarded and 1 was repeated]). Spot pI=6.8. MW=28 kDa. | | | | | | | | | | |
| H6 | ribosome biogenesis protein NSA2-like | OLP85990.1 | | dinoflagellate | 2 | 44 | 15 | Sym | comp89857_c0_seq1 | 2>36 |
| H6 | pentatricopeptide repeat-containing protein | OLQ13535.1 | | dinoflagellate | 2 | 43 | 9 | Sym | comp306229_c0_seq1 | H2>C2, H>C |
| H6 | voltage-dependent T-type calcium channel subunit α-1H | OLP96691.1 | | dinoflagellate | 2 | 41 | 8 | Sym | comp99263_c0_seq1 | 2>36, H>C |
| H6 | nucleolar protein of 40 kDa | OLP94620 | | dinoflagellate | 3 | 36 | 13 | Sym | comp106753_c0_seq1 | 2>36, H36>C36 |
| H6 | peptidylprolyl isomerase D^e^ | OLQ06903 | | dinoflagellate | 2 | 25 | 2 | Sym | comp122885_c0_seq4 | H2>C2, 2>36, H>C |

^a^Protein concentration affected by temperature treatment in a study undertaken with the con-familial coral *Seriatopora hystrix* [32]. ^b^Weak homology to an alveolate sequence (could also be of *Symbiodinium* origin). ^c^Down-regulated at high temperature in *P. acuta* larvae [20]. ^d^potential pseudogene. ^e^closely related paralog found in spot H3.
