# Supplementary material for: The proteomic response of the reef coral Pocillopora acuta to experimentally elevated temperatures
Source: PLoS One. 2018 Jan 31;13(1):e0192001. doi: 10.1371/journal.pone.0192001 (PMC5792016; doi:10.1371/journal.pone.0192001)
Supplement: S2 Table — Proteins highlighted in blue and grey also differed in concentration between treatments at the two- and eight-week sampling times, respectively (Table 2). Please see the S5 Table for hypothetical functions and peptide sequences. “C” and “H” in the “Spot” column correspond to spots removed from the control and high-temperature treatment gels, respectively. AA = amino acids. DCP = differentially concentrated protein. kDa = kilodalton. MW = molecular weight. pI = isoelectric point. Sym = Symbiodinium. (DOCX) [file pone.0192001.s003.docx]

**S2 table. Proteins whose concentrations differed between temperature treatments at the four-week sampling time.** Proteins highlighted in blue and grey also differed in concentration between temperature treatments at the two- and eight-week sampling times, respectively (Table 2). Please see the S5 table for hypothetical functions and peptide sequences. When two accession numbers have been included for the same protein, the top and bottom correspond to the top hit acquired upon BLASTing the sequence of the mRNA encoding the sequenced peptide and the top hit acquired upon BLASTing the peptide sequence itself, respectively. “C” and “H” in the “Spot” column correspond to spots removed from the control and high-temperature treatment gels, respectively. AA=amino acids. DCP=differentially concentrated protein. kDa=kilodalton. MW=molecular weight. pI=isoelectric point. Sym=*Symbiodinium*.

| **Spot** | **Protein** | **NCBI accession of top hit** | **Top hit taxon** | **# Unique peptides** | **#AA se-quenced** | **% Co- verage** | **Com-part-ment** | ***Pocillopora acuta* transcriptome contig** |
| --- | --- | --- | --- | --- | --- | --- | --- | --- |
| **Spot C1: higher concentration in control samples** (n=3 [1 was discarded]). Spot pI=4.3. MW=28 kDa. | | | | | | | | |
| C1 | pentraxin^a^ | XP_001621255  XP_022787255 | coral | 4 | 69 | 15 | host | comp40757_c0_seq1 |
| C1 | trichohyalin^b^ | XP_015747738 | coral | 2 | 36 | 14 | host | comp119340_c1_seq4 |
| C1 | plexin | XP_015777577  XP_022785730 | coral | 2 | 46 | 2 | host | comp120799_c0_seq4 |
| **Spot C2: higher concentration in control samples** (n=2 [2 were discarded]). Spot pI=4.5. MW=11 kDa. | | | | | | | | |
| C2 | avidin | ELT93730.1 | worm | 2 | 32 | 23 | host | contig12534 |
| C2 | hypothetical protein | XP_015765192.1 | anemone | 1 | 31 | 4 | host | comp120156_c1_seq1 |
| **Spot C3: higher concentration in control samples** (n=1 [2 were discarded]). Spot pI=5.4. MW=32 kDa. | | | | | | | | |
| C3 | chloroplast oxygen-evolving enhancer | AGL79735 | dinoflagellate | 2 | 33 | 9 | Sym | comp113817_c0_seq2 |
| **Spot C4: higher concentration in control samples** (n=2 [2 were discarded]). Spot pI=5.6. MW=32 kDa. | | | | | |  |  |  |
| C4 | actin | AFG33344.1 | insect | 2 | 32 | 37 | host | comp603506_c0_seq1 |
| C4 | actin (different paralog from previous) | AGG36337 | coral | 5 | 44 | 12 | host | contig10370 |
| **Spot H5: higher concentration in high temperature samples** (n=0 [2 were discarded]). Spot pI=6.1. MW=18 kDa: No unique DCPs identified. | | | | | | | | |
| **Spot H6: higher concentration in high temperature samples** (n=0 [2 were discarded]). Spot pI=5.8. MW=22 kDa: No unique DCPs identified. | | | | | | | | |
| **Spot H7: higher concentration in high temperature samples** (n=2 [2 were discarded]). Spot pI=5.7. MW=35 kDa. | | | | | | | | |
| H7 | RNA-directed DNA polymerase from mobile element jockey-like | XP_015749727.1  PFX22074.1 | coral | 2 | 35 | 4 | host | contig9441 |
| H7-8 | peridinin-chlorophyll A binding protein | PCP_SYMSP  AFH88373.1 | dinoflagellate | 12 | 130 | 34 | Sym | contig14131 |
| **Spot H8: higher concentration in high temperature samples** (n=2 [1 was discarded and 1 was repeated]). Spot pI=5.9 MW=35 kDa. | | | | | | | | |
| H8 | NF-kappa-B inhibitor-like protein 1 isoform X2 | XP_020610253  XP_022794399.1 | coral | 2 | 35 | 10 | host | comp115480_c0_seq1 |
| H8 | hypothetical protein^c^ | KXJ05512 | anemone | 2 | 20 | 7 | host | contig5078 |

^a^Protein also documented at higher concentration in control temperature samples at the two-week sampling time. ^b^Protein also documented at higher concentration in high temperature samples at the eight-week sampling time. ^c^Protein also documented at higher concentration in high temperature samples at the two-week sampling time.
