# Supplementary material for: The proteomic response of the reef coral Pocillopora acuta to experimentally elevated temperatures
Source: PLoS One. 2018 Jan 31;13(1):e0192001. doi: 10.1371/journal.pone.0192001 (PMC5792016; doi:10.1371/journal.pone.0192001)
Supplement: S3 Table — Underlined spots represent uniquely synthesized proteins (see Fig 1 and Table 1.). Proteins highlighted in yellow and grey also differed in concentration between treatments at the two- and four-week sampling times, respectively (Table 2), and those highlighted in red were hypothesized to be involved in the stress response. Please see the S6 Table for hypothetical functions and peptide sequences. “C” and “H” in the “Spot” column correspond to spots removed from the control and high-temperature treatment gels, respectively. AA = amino acids. kDa = kilodalton. MW = molecular weight. pI = isoelectric point. Sym = Symbiodinium. (DOCX) [file pone.0192001.s004.docx]

**S3 table.** **Proteins whose concentrations differed between temperature treatments at the eight-week sampling time.** Underlined spots represent uniquely synthesized proteins (see Fig 1 and Table 1.). Proteins highlighted in yellow and grey also differed in concentration between treatments at the two- and four-week sampling times, respectively (Table 2), and those highlighted in red were hypothesized to be involved in the stress response. Please see the S6 table for hypothetical functions and peptide sequences. When two accession numbers have been included for the same protein, the top and bottom correspond to the top hit acquired upon BLASTing the sequence of the mRNA encoding the sequenced peptide and the top hit acquired upon BLASTing the peptide sequence itself, respectively. “C” and “H” in the “Spot” column correspond to spots removed from the control and high temperature treatment gels, respectively. AA=amino acids. kDa=kilodalton. MW=molecular weight. pI=isoelectric point. Sym=*Symbiodinium*.

| **Spot** | **Protein** | **NCBI accession of top hit** | **Top hit taxon** | **# Unique peptides** | **#AA se-quenced** | **% Co-verage** | **Com-part-ment** | ***Pocillopora acuta* transcriptome contig** |
| --- | --- | --- | --- | --- | --- | --- | --- | --- |
| **Spot C1: only translated by control samples** (n=5 [2 were discarded]). Spot pI=5.1. MW=27 kDa. | | | | | | | | |
| C1 | rho GDP-dissociation inhibitor 1-like | XP_020626162  XP_022799157 | coral | 2 | 32 | 16 | host | Contig413 |
| C1 | hypothetical protein | scaffold1082.1^a^ | dinoflagellate | 1 | 25 | 24 | Sym | mira_454_illumina_rep_c521 |
| C1 | fucoxanthin-chlorophyll a-c binding protein F | OLP83973  OLP78220 | dinoflagellate | 4 | 87 | 21 | Sym | comp115079_c0_seq1 |
| C1 | hypothetical protein | scaffold4958.1^a^ | dinoflagellate | 2 | 49 | 5 | Sym | comp108367_c0_seq1 |
| C1 | metal transporter Nramp3 | OLP79210 | dinoflagellate | 3 | 57 | 4 | Sym | comp110783_c0_seq1 |
| **Spot C2: higher concentration in control samples** (n=1 [4 were discarded]). Spot pI=5.5. MW=32 kDa. | | | | | | | | |
| C2 | centromere-associated protein E-like isoform X1 | XP_015764703 | coral | 2 | 36 | 1 | host | comp123903_c0_seq1 |
| **Spot H1: only translated by high temperature samples** (n=4 [3 were discarded]). Spot pI=5.9. MW=43 kDa. | | | | | | | | |
| H1 | trichohyalin | XP_015747738 | coral | 2 | 42 | 17 | host | comp119340_c1_seq4 |
| H1 | hypothetical protein | OLP93115 | dinoflagellate | 2 | 25 | 2 | Sym | comp118297_c0_seq1 |
| H1 | hypothetical protein | No hit | unknown | 2 | 26 | 20 | unknown | comp108045_c3_seq1 |
| H1 | VWF domain-containing protein | WP_008002149 | bacteria | 3 | 31 | 12 | unknown | Contig14091 |
| **Spot H2: only translated by high temperature samples** (n=3 [3 were discarded and 2 were repeated]). Spot pI=6.2. MW=45 kDa. | | | | | | |  |  |
| H2 | abhydrolase | XP_015777141  XP_022805068 | coral | 10 | 113 | 34 | host | Contig269 |
| H2 | chromodomain-helicase-DNA-binding protein 1-like | XP_020631936  XP_022780357 | coral | 2 | 39 | 3 | host | Contig6712 |
| H2 | hypothetical protein | OLQ01765 | dinoflagellate | 1 | 28 | 5 | Sym | mira_454_illumina_rep_c25 |
| **Spot H3: only translated by high temperature samples** (n=5 [2 were discarded and 1 was repeated]). Spot pI=6.3. MW=40 kDa. | | | | | | | | |
| H3 | retrovirus-related Pol polyprotein from transposon 17.6 | KXJ08983  PFX14876 | anemone & coral | 2 | 27 | 36 | host | comp987274_c0_seq1 |
| H3 | guanine nucleotide-binding protein G(I)/G(S)/G(T) subunit beta-1 | XP_020630575 XP_022793483 | coral | 7 | 94 | 27 | host | contig931 |
| H3 | stabilizer of axonemal microtubules 2-like | XP_020603745  XP_022797002 | coral | 2 | 59 | 13 | host | comp121775_c0_seq6 |
| H3 | glycerol-3-phosphate dehydrogenase | XP_015772268  XP_022782545 | coral | 3 | 45 | 12 | host | Contig6731-33 |
| H3 | concanavalin A-like lectin/glucanase superfamily | XP_015754876 | coral | 2 | 57 | 4 | host | comp123756_c0_seq3 |
| **Spot H4: higher concentration in high temperature samples** (n=4 [1 was discarded and 1 was repeated]). Spot pI=6.2. MW=29 kDa. | | | | | | | | |
| H4 | glycosyltransferase-like domain-containing protein 1 isoform X2 | XP_015770652 | coral | 5 | 76 | 33 | host | Contig3845 |
| H4 | trichohyalin-like | XP_020610789 | coral | 2 | 47 | 11 | host | comp119772_c3_seq1 |
| H4 | protein w/ DNAJ and WW domains | OLP80612 | dinoflagellate | 2 | 18 | 8 | Sym | comp109287_c2_seq1 |
| H4 | hypothetical protein | no hit | no hit | 2 | 52 | 18 | unknown | mira_454_illumina_rep_c22256 |
| **Spot H5: higher concentration in high temperature samples** (n=2 [2 were discarded and 2 were repeated]). Spot pI=6.0. MW=27 kDa. | | | | | | | | |
| H5 | peroxiredoxin-6-like | XP_020630207  XP_022795312 | coral | 10 | 88 | 40 | host | Contig1472 |
| H5 | hypothetical protein | XP_020603766 | coral | 5 | 68 | 37 | host | Contig3736 |
| **Spot H6: higher concentration in high temperature samples** (n=1 [1 was discarded and 1 was repeated]). Spot pI=6.0. MW=29 kDa. | | | | | | | | |
| H6 | endonuclease | XP_015775232.1 | coral | 3 | 33 | 10 | host | Contig11635 |

^a^Compartment of origin inferred from significant alignment to unpublished *Symbiodinium* genome (http://marinegenomics.oist.jp/symb).
