# Supplementary material for: The proteomic response of the reef coral Pocillopora acuta to experimentally elevated temperatures
Source: PLoS One. 2018 Jan 31;13(1):e0192001. doi: 10.1371/journal.pone.0192001 (PMC5792016; doi:10.1371/journal.pone.0192001)
Supplement: S4 Table — The 13 and 25 unique proteins whose concentrations were higher in samples of the control (C) and high temperature (H) treatments, respectively, at the two-week sampling time were included. Additional details of the sequenced proteins can be found in the S1 Table. Although multiple missed cleavages exist in certain peptide sequences (more than the two allowed by the MS-GF+ script [described in the supplemental methods section of the S1 file]), these peptides were nevertheless included provided that they were either 1) 15 or more amino acids (AA) in length or 2) paired with one or more additional peptides that mapped to the same reference protein (whose collective length summed to 15 or more AA). The compartment of origin has been mentioned next to the spot number when it could be determined. Of the 38 proteins identified by BLAST analysis of the top hit contig (mRNA) derived from MS-SCAN analysis of the Pocillopora acuta-Symbiodinium (“Sym”) transcriptome (see the S1 Table for contig accession numbers.), the identities of 12 were further verified by directly BLASTing the MS-SCAN-derived peptide sequences against the Stylophora pistillata (n = 8) and Symbiodinium (clade B1) genomes (n = 4). (DOCX) [file pone.0192001.s005.docx]

**S4 table. Peptide sequences for proteins whose concentrations differed between temperature treatments at the two-week sampling time**. The 13 and 25 unique proteins whose concentrations were higher in samples of the control (C) and high temperature (H) treatments, respectively, at the two-week sampling time were included. Additional details of the sequenced proteins can be found in the S1 table. Although multiple missed cleavages exist in certain peptide sequences (more than the two allowed by the MS-GF+ script [described in the supplemental methods section of the S1 file]), these peptides were nevertheless included provided that they were either 1) 15 or more amino acids (AA) in length or 2) paired with one or more additional peptides that mapped to the same reference protein (whose collective length summed to 15 or more AA). The compartment of origin has been mentioned next to the spot number when it could be determined. Of the 38 proteins identified by BLAST analysis of the top hit contig (mRNA) derived from MS-SCAN analysis of the *Pocillopora acuta-Symbiodinium* (“Sym”) transcriptome (see the S1 table for contig accession numbers.), the identities of 12 were further verified by directly BLASTing the peptide sequences against the *Stylophora pistillata* (n=8) and *Symbiodinium* (clade B1) genomes (n=4).

| **Spot(s)** | **Protein** | **Sequence** | **Function** | **Notes** |
| --- | --- | --- | --- | --- |
| C1-2-host | pentraxin^a^ | YADQDLPSDGLFHHFCFTWSNTNGDYKFWIDGEVVGSGSDLYKGGTIEKGGTVVIGQDQDEVGGDFDPR | immunity | 4 sequenced peptides overlapped |
| C1-host | transcription factor death-induced obliterator-1 | SGRHWHEDEHRRDRHAQPIDERPRYQERRDER | transcription factor | 2 sequenced peptides overlapped |
| C1-Sym | protein kinase UbiB | PPDAEDQDKHLPDGLQLVDLTKESK | protein kinase |  |
|  |  | GRGKGKGKGARNFKRVRDRLKK |  |  |
| C1-Sym | STI1-like protein | DAYLDPTKGEEHREKGNEFFKEKKYAEAKAEYDEGIKRNPK | stress response | 2 sequenced peptides overlapped |
| C1-Sym | myosin-6^b^ | KYCADLENLLETLNTCHLHYI | actin-based motility |  |
|  |  | LFLKAGQLKALEDMRTEGAEADPER |  |  |
| C2-host | WSC domain | DRHRNPRPLPNYLMNDRDVFHK | unknown |  |
|  |  | LKLRQLVASMRCEMIELWAKKSTTKWTHPAQCLR |  |  |
| C2-host | cadherin EGF LAG seven-pass G-type receptor 2^a^ | EKSSDNHEGDDDVEDDEKQTSHRGNTHKK | receptor/signaling |  |
|  |  | VYNADNDDDDQYYGGEGNQEYQGRNHYK |  |  |
| C2-Sym | pre-mRNA splicing factor  SLU7-A^b^ | RDRWNGFQPDDYKQVIEDWERVEAERR | gene expression/splicing | 2 sequenced peptides overlapped |
| C3-host | hypothetical protein | RRKIIIIKKLLYNYVEHIKLLIALRVK | unknown | 2 sequenced peptides overlapped |
|  |  | YTDGQAQAKRLCKGVTKENTNLRK |  |  |
| C3-host | Pao retrotransposon peptidase | AINCENVQSIEERKK | unknown/various |  |
|  |  | ANDCKSRNTCKICDGKHHTSICDRKER |  |  |
| C3-host | spectrin alpha chain^a^ | SYIPPPTPTFEKDRIRALKNERINSQKK | cytoskeleton | 2 sequenced peptides overlapped |
|  |  | VLVKRKVKKKVKIQKQR |  |  |
| C3-Sym | adenylate kinase | WAIFDRAMEETTESLRER | protein kinase |  |
|  |  | EEFLKGCALPSDLKEMTEK |  |  |
| C3 | hypothetical protein | KKKRLRKKIKKKSRKKKRKRKK | unknown | 2 sequenced peptides overlapped |
| H1-host | hypothetical protein | KKKKKKRRRKKKRKKKEKKK | unknown | 2 sequenced peptides overlapped |
| H1, H3-host | low-density lipoprotein receptor-related protein 4^a^ | CVSNDFLCDMEDDCGDR | receptor/transport |  |
|  |  | CDGNDDCGDESDERNCQTTSVCKANEFR |  |  |
| H1-host | serine/arginine repetitive matrix protein 1^a^ | SRASRRSPSSSPEREVKPRSRAAKQSSPASPETR | gene expression/splicing | 2 sequenced peptides overlapped |
| H1-Sym | Rec10 / Red1 | KAKKVKKAKKVKKAKKSKKDDSDKKGKKKHSK | meiosis | 2 sequenced peptides overlapped |
| H1-bacteria | VWA domain-containing protein | VLILTFEPSSGGKCCDEEDDDDDDDDDDRKR | unknown | 2 sequenced peptides overlapped |
| H2-host | hypothetical protein | TQLLLEVEEQLNERCPCGNCGMFDRDR | unknown |  |
|  |  | ELRKLVGLHHDVQLLCLELHVKQFVGERLLPLLEK |  |  |
| H2-host | histone-lysine N-methyltransferase SETD1B-like^a^ | DDNAKTTNKLIESFRRESMRTSSPPSKSR | gene expression | 2 sequenced peptides overlapped |
| H2-Sym | ribulose-1,5-bisphosphate carboxylase/oxygenase^b^ | GTTNGGLVVGTIIKPK | photosynthesis |  |
|  |  | GYTAFVHTK |  |  |
|  |  | LPAFFENLGHSNVILTAGGGSFGHK |  |  |
|  |  | AGQFGNISLSDGVIEYAK |  |  |
|  |  | GAFLTFQK |  |  |
| H2-3-Sym | DNAJ and WW domains | KKKKKDKKKKKKKKKSKK | stress response | 2 sequenced peptides overlapped |
| H2 | hypothetical protein | KVMRRRKRSIKRRRRIRRKKMMAIKNKRKKRSQKRRK | unknown | 2 sequenced peptides overlapped |
| H3-host | golgin subfamily B member 1^a^ | TIEGLMDRLDAQKRLIDSR | unknown |  |
|  |  | DELSKANSELEKVKSVNERLK |  |  |
| H3-Sym | peptidylprolyl isomerase D | EKEEELERKQKEEAQKRR | stress response | 2 sequenced peptides overlapped |
| H3 | hypothetical protein | KKKRKGKIVHIPLRRLRAILLVRVKIIKLR | unknown | 2 sequenced peptides overlapped |
| H3 | hypothetical protein | ESRGAEERSRDRDNRKKERREK | unknown |  |
|  |  | RSPRRSRSRKRSRSRPKASRRDSRARK |  |  |
| H4-Sym | E3 ubiquitin protein ligase | DNEDDCDHIFCSNPECR | stress response |  |
|  |  | KVVEVHGNHHHKPKCPYYFEFK |  |  |
| H4 | hypothetical protein | QQELIQKQKEELKQVKEQK | unknown | 2 sequenced peptides overlapped |
| H4 | hypothetical protein | IRRRRLRRLPRIRRRVRRVRRIRR | unknown | 2 sequenced peptides overlapped |
| H5-6-host | beta-gamma crystallin | ADVTSGTWIFYTHANNNDKEAGAGPSNYK | stress response |  |
|  |  | GEGVSSAIVLSK |  |  |
|  |  | FAIFTKPNYQGLQQQLEPDTR |  |  |
| H5-host | sacsin^a^ | LLQMFLEGAESLLLFSQNILRVGIYNLPR | stress response |  |
|  |  | LYKFCRFEEISNAQRRNREDR |  |  |
| H5 | hypothetical protein | ERSERQRATQLCKAQDEELKRRER | unknown | 2 sequenced peptides overlapped |
| H6-Sym | ribosome biogenesis protein NSA2-like^b^ | EPKKISKTARKLRGIKAKLFTKKRYTEKATMKKTIKAHQEKDAK | ribosome | 2 sequenced peptides overlapped |
| H6-Sym | pentatricopeptide repeat-containing protein | MWWDALLEVFEMKNK | unknown |  |
|  |  | YTSIVFNALACCLRCDAEKVSACQLLQR |  |  |
| H6-Sym | voltage-dependent T-type calcium channel subunit α-1H | NCYANKCSHCKNLRSFTRPSFTIEELAMRSTTLTRNFRYQK | transport | 2 sequenced peptides overlapped |
| H6-Sym | nucleolar protein of 40 kDa | AVMEEVKRMKKESKLKKKFLKLENKRKKKLQKKLKK | unknown | 3 sequenced peptides overlapped |
| H6-Sym | peptidylprolyl isomerase D^c^ | RRSHARPGFRGNRGMVADRVQAERR | stress response | 2 sequenced peptides overlapped |

^a^Protein identity corroborated by directly BLASTing the peptide sequence against the *Stylophora pistillata* genome.

^b^Protein identity corroborated by directly BLASTing the peptide sequence against the *Symbiodinium* (clade B1) genome.

^c^Different from paralog identified in spot H3.
