# Supplementary material for: The proteomic response of the reef coral Pocillopora acuta to experimentally elevated temperatures
Source: PLoS One. 2018 Jan 31;13(1):e0192001. doi: 10.1371/journal.pone.0192001 (PMC5792016; doi:10.1371/journal.pone.0192001)
Supplement: S5 Table — The eight and four proteins whose concentrations were higher in samples of the control (C) and high temperature (H) treatments, respectively, at the four-week sampling time were included. Additional details of the sequenced proteins can be found in the S2 Table. Although multiple missed cleavages exist in certain peptide sequences (more than the two allowed by the MS-GF+ script [desribed in the supplemental methods outlined in the S1 file]), these peptides were nevertheless included provided that they were either 1) 15 or more amino acids (AA) in length or 2) paired with one or more additional peptides that mapped to the same reference protein (whose collective length summed to 15 or more AA). No unique, differentially concentrated proteins were identified from spots H5 and H6. The compartment of origin has been included next to the spot number. Of the 12 proteins identified by BLAST analysis of the top hit contig derived from MS-SCAN analysis of the Pocillopora acuta-Symbiodinium (“Sym”) transcriptome (see the S2 Table for contig accession numbers.), the identies of half were further verified by directly BLASTing the MS-SCAN-derived peptide sequences against the Stylophora pistillata (n = 4) and Symbiodinium (clade B1) genomes (n = 2). (DOCX) [file pone.0192001.s006.docx]

**S5 table. Peptide sequences for proteins whose concentrations differed between temperature treatments at the four-week sampling time**. The eight and four proteins whose concentrations were higher in samples of the control (C) and high temperature (H) treatments, respectively, at the four-week sampling time were included. Additional details of the sequenced proteins can be found in the S2 table. Although multiple missed cleavages exist in certain peptide sequences (more than the two allowed by the MS-GF+ script [desribed in the supplemental methods outlined in the S1 file]), these peptides were nevertheless included provided that they were either 1) 15 or more amino acids (AA) in length or 2) paired with one or more additional peptides that mapped to the same reference protein (whose collective length summed to 15 or more AA). No unique, differentially concentrated proteins were identified from spots H5 and H6. The compartment of origin has been included next to the spot number. Of the 12 proteins identified by BLAST analysis of the top hit contig derived from MS-SCAN analysis of the *Pocillopora acuta-Symbiodinium* (“Sym”) transcriptome (see the S2 table for contig accession numbers.), the identies of half were further verified by directly BLASTing the peptide sequences against the *Stylophora pistillata* (n=4) and *Symbiodinium* (clade B1) genomes (n=2).

| **Spot(s)** | **Protein** | **Sequence** | **Function** | **Notes** |  |
| --- | --- | --- | --- | --- | --- |
| C1-host | pentraxin^a,b^ | FWIDGEVVGSGSDLYK | immunity | 2 sequenced peptides overlapped |  |
|  |  | GGTVVIGQDQDEVGGDFDPR |  |  |  |
| C1-host | trichohyalin^c^ | RRRRRRRRRRRRKLRRYGRRRPRRGRRRRRGRRGRR | structural | 2 sequenced peptides overlapped |  |
| C1-host | plexin^b^ | LCQEDEVYSSYMELPLDCK | signal tranduction/reception |  |  |
|  |  | FFCDFSMEHSQEFDGDSALQELYDYVR |  |  |  |
| C2-host | avidin | GLYANGAPGSEVLLEK | reproduction |  |  |
|  |  | DVPATLGFVVNFEGGK |  |  |  |
| C2-host | hypothetical protein | FCDLEDRVFEKQQSRK | unknown |  |  |
|  |  | MRPSTCPTSSSRWNR |  |  |  |
| C3-Sym | chloroplast oxygen-evolving enhancer^d^ | SFAVEIDTDNGKEFATTK | photosynthesis |  |  |
|  |  | VNQELGEIGGVFVSK |  |  |  |
| C4-host | actin^e^ | FEQEMATAAASTSLEKSYELPDGQVITIGNER | cytoskeleton | 2 sequenced peptides overlapped |  |
| C4-host | actin (different from previous paralog) | AVFPSIVGR | cytoskeleton | 2 sequenced peptides overlapped |  |
|  |  | VAPEEHPVLLTEAPLNPK |  |  |  |
|  |  | GYSFTTTAER |  |  |  |
|  |  | FTTTAER |  |  |  |
| H7-host | RNA-directed DNA polymerase from mobile element jockey-like^b^ | WLRHRNVVLVGDFNSDYFR | gene expression/splicing |  |  |
|  |  | GHSTELLLVKITDDWR |  |  |  |
| H7-8-Sym | peridinin-chlorophyll A binding protein^d^ | KLGDASYSFAK | photosynthesis | multiple peptide |  |
|  |  | LLKDAAEAHHKAIGSISGPNGVTSR |  | sequences |  |
|  |  | SLVNGPDAEKAYQGFLEFKDVVEKNQVATASAPAVVPSGDKIGEAAKALSDASYPFIK | | overlapped | overlapped |
|  |  | AIGSIDATGVTSAADYEAVNAAIGR |  |  |  |
|  |  | VNPLDAVAAAK |  |  |  |
| H8-host | NF-kappa-B inhibitor-like protein 1 isoform X2^b^ | EELEAERKERWHKEHEEK | immunity |  |  |
|  |  | ETYDEWADRITYERRQK |  |  |  |
| H8-host | hypothetical protein^a^ | KKKKKKRRRKKKRKKKEKKK | unknown | 2 sequenced peptides overlapped |  |

^a^Also identified at the two-week sampling time. ^b^Protein identity corroborated by direct BLAST of the peptide sequence against the *Stylophora pistillata* genome. ^c^Also identified at the eight-week sampling time. ^d^Protein identity corroborated by direct BLAST of the peptide sequence against the *Symbiodinium* (clade B1) genome. ^e^Aligns significantly to plant and bacterial actins, as well; interpret cautiously.
