# Supplementary material for: The proteomic response of the reef coral Pocillopora acuta to experimentally elevated temperatures
Source: PLoS One. 2018 Jan 31;13(1):e0192001. doi: 10.1371/journal.pone.0192001 (PMC5792016; doi:10.1371/journal.pone.0192001)
Supplement: S6 Table — The 6 and 19 proteins whose concentrations were higher in samples of the control (C) and high (H) temperature treatments, respectively, at the eight-week sampling time were included. Additional details of the sequenced proteins can be found in the S3 Table. Although multiple missed cleavages exist in certain peptide sequences (more than the two allowed by the MS-GF+ script [described in the supplemental methods outlined in the S1 file]), these peptides were nevertheless included provided that they were either 1) 15 or more amino acids (AA) in length or 2) paired with one or more additional peptides that mapped to the same reference protein (whose collective length summed to 15 or more AA). The lysine- and arginine-rich nature of the Pocillopora acuta and Symbiodinium (“Sym”) proteomes of this study, as well as another carried out with the con-familial pocilloporid coral Seriatopora hystrix [32], suggests that alternative protein digest enzymes may serve better for future proteomic analyses of pocilloporid corals. The compartment of origin has been included next to the spot number when it could be determined. Of the 25 proteins identified by BLAST analysis of the top hit contig derived from MS-SCAN analysis of the P. acuta-Symbiodinium transcriptome (see the S3 Table for contig accession numbers.), the identities of 11 were further verified by directly BLASTing the MS-SCAN-derived peptide sequences against the Stylophora pistillata (n = 8) and Symbiodinium (clade B1) genomes (n = 3). bact = bacterial. (DOC) [file pone.0192001.s007.doc]

**S6 table. Peptide sequences for proteins whose concentrations differed between temperature treatments at the eight-week sampling time**. The 6 and 19 proteins whose concentrations were higher in samples of the control (C) and high (H) temperature treatments, respectively, at the eight-week sampling time were included. Additional details of the sequenced proteins can be found in the S3 table. Although multiple missed cleavages exist in certain peptide sequences (more than the two allowed by the MS-GF+ script [described in the supplemental methods outlined in the S1 file]), these peptides were nevertheless included provided that they were either 1) 15 or more amino acids (AA) in length or 2) paired with one or more additional peptides that mapped to the same reference protein (whose collective length summed to 15 or more AA). The lysine- and arginine-rich nature of the *Pocillopora acuta* and *Symbiodinium* (“Sym”) proteomes of this study, as well as another carried out with the con-familial pocilloporid coral *Seriatopora hystrix* [32], suggests that alternative protein digest enzymes may serve better for future proteomic analyses of pocilloporid corals. The compartment of origin has been included next to the spot number when it could be determined. Of the 25 proteins identified by BLAST analysis of the top hit contig derived from MS-SCAN analysis of the *P. acuta-Symbiodinium* transcriptome (see the S3 table for contig accession numbers.), the identities of 11 were further verified by directly BLASTing the peptide sequences against the *Stylophora pistillata* (n=8) and *Symbiodinium* (clade B1) genomes (n=3). bact=bacterial.

| **Spot(s)** | **Protein** | **Sequence** | **Function** | **Notes** |
| --- | --- | --- | --- | --- |
| C1-host | rho GDP-dissociation inhibitor 1-like^a^ | TLQEIQTLDADDESLVR | protein homeostasis |  |
|  |  | ADPQEYLTPCDEAPK |  |  |
| C1-Sym | hypothetical protein | RPPALGPQQQCKKRPPALRPQQQCK | unknown |  |
| C1-Sym | fucoxanthin-chlorophyll a-c binding protein F^b^ | ELGVQEPIGFWDPLGL SADKDEATFK | photosynthesis |  |
|  |  | LPVLGGAQIIAFAGLIETTGFFQAASTTDGR |  |  |
|  |  | DSTETGEPGNYGVGFPTFLGK |  |  |
|  |  | LAAELANGR |  |  |
| C1-Sym | hypothetical protein^b^ | KHYCCEKEGKGCYDCNAGLANWDRGWSDKKK | unknown |  |
|  |  | RHYCCTMHHIGCDLYHCR |  |  |
| C1-Sym | metal transporter Nramp3 | KRAVRLEQLDREFREQKAMGR | transport |  |
|  |  | ESSCLENLFACLGRKDRRPRR |  |  |
|  |  | QNEALQTQVAGMRNR |  |  |
| C2-host | centromere-associated protein E-like isoform X1 | EMEERLSGANEQEER | cell cycle |  |
|  |  | KDLNENKCSIEARFKQFKEEK |  |  |
| H1-host | trichohyalin^c^ | QPRRLSRRQRRRMRRRR | cell structure |  |
|  |  | RRRRRRRRRRRRRRRRRRKLRRYGR |  |  |
| H1-Sym | hypothetical protein | RRRRSRRRRSRRRRRSRRRRSRRRR | unknown | 2 sequenced peptides overlapped |
| H1 | hypothetical protein | KKKKDKKDKKKKKEKKKKEKKEKKEKK | unknown | 2 sequenced peptides overlapped |
| H1-bact | hypothetical bacterial protein (VWF domain-containing protein)^d^ | VLILTFEPSSGGKCCDEEDDDDDDDDDDRKR | unknown | 3 sequenced peptides overlapped |
| H2-host | abhydrolase^a^ | FFQHEDIVHLLDRFVIYHVDAPGQESKAENLAPDYQYPSITK | metabolism | numerous sequenced peptides overlapped |
|  |  | VAGLVVVEPGISAASFK |  |  |
|  |  | GFTEDTIK |  |  |
|  |  | TGVLVVTGEHSPHRKNTEDKFYYGDVLK |  |  |
|  |  | SLLTPDCGASVLEEKPEK |  |  |
| H2-host | chromodomain-helicase-DNA-binding protein 1-like^a,e^ | EDVEGFSDAEIRRFVKSYKK | DNA repair/ |  |
|  |  | AVAEYEEKLKEDPNFDGKK | transcription |  |
| H2-Sym | hypothetical protein^b^ | NVSGEGDEDDLAEKLEETQRLLEDARDR | unknown |  |
| H3-host | retrovirus-related Pol polyprotein from transposon 17.6^a^ | IQNDVELVKKRVIETDIVRKRKLLKRR | unknown | 2 sequenced peptides overlapped |
| H3-host | guanine nucleotide-binding protein G(I)/G(S)/G(T) subunit beta-1^a^ | KAVCDTSLTQVTHNIEAVGR | signaling |  |
|  |  | NLVSASQDGK |  |  |
|  |  | VHIIPLR |  |  |
|  |  | ELPGHTGYLSCCR |  |  |
|  |  | SFVSGACDASAK |  |  |
|  |  | QTFTGHESDINAVAYFPNGYAFATGSDDATCR |  | 2 sequenced peptides overlapped |
| H3-host | stabilizer of axonemal microtubules 2-like^a^ | HDYPGTVVPPATSAKPLATFHAHESPFDGSTVHQDTYR | cytoskeleton |  |
|  |  | SGRPDEYRPPSGKFHGESTMR |  |  |
| H3-host | glycerol-3-phosphate dehydrogenase^a^ | KLTEIINTEHENVK | metabolism |  |
|  |  | LPDNIVANPDVVDAAKDADILVFVVPHQFVK |  | 2 sequenced peptides overlapped |
| H3-host | concanavalin A-like lectin/glucanase superfamily | RAKRKQRRVTKTRKLKLRIIKRIRLLKVLKR | cell membrane binding |  |
|  |  | MRLLIRQRMLQIKRKLALRQRILRSR |  |  |
| H4-host | glycosyltransferase-like domain-containing protein 1 | YFTGGLHTLHASSTKEEIEECYDK | metabolism |  |
|  |  | GYTNVDALDVTQK |  |  |
|  |  | FICAHLTEAR |  |  |
|  |  | VISDGGVIYFNVR |  |  |
|  |  | EEQVPHYNIEDPALPK |  |  |
| H4-host | trichohyalin-like | LRIRYGMRWRIVRGKLPKGRRQRRKRR | cell structure |  |
|  |  | IYIRSRPQFMIKRRWRRARR |  |  |
| H4-Sym | protein w/ DNAJ and WW domains^d^ | KKKKKDKKKKKKKKKSKK | stress response | 2 sequenced peptides overlapped |
| H4 | hypothetical protein | RKTPLNRTKRRRRSRMRKKRKVRLQNLRRRSMRR | unknown |  |
|  |  | RRRVRRQRKTLPNVRRRR |  |  |
| H5-host | peroxiredoxin-6-like^a^ | VNLGDVFPDFNADTSK | stress response | several sequenced peptides overlapped |
|  |  | DIQGYSGLSGDFPYPIIADEKR |  |  |
|  |  | DAEGLPLTARAVFIIGPDKK |  |  |
|  |  | LSLLYPATTGRNFDEILRVIDSLQLTAIKK |  |  |
| H5-host | hypothetical protein | SGNVLAINQDDDTVIAK | unknown |  |
|  |  | ILDEVKEDSPEILFYK |  |  |
|  |  | VPGSELYYFTSFLK |  |  |
|  |  | IIGFDPEGKQINTTEVQPDKR |  | 2 sequenced peptides overlapped |
| H6-host | endonuclease | KVGVLKKIKKIKNIKKIKRIKKIKKKKIKKTNK | DNA repair | 3 sequenced peptides overlapped |

^a^Protein identity corroborated by direct BLAST of the peptide sequence against the *Stylophora pistillata* genome. ^b^Protein identity corroborated by direct BLAST of the peptide sequence against the *Symbiodinium* (clade B1) genome. ^c^Also identified at the four-week sampling time. ^d^Also identified at the two-week sampling time. ^e^Protein concentration affected by temperature treatment in a study undertaken with the con-familial coral *Seriatopora hystrix* [32].
